# Efficacy and safety of gabapentinoid combination therapy versus monotherapy for the treatment of neuropathic pain

**DOI:** 10.3389/fphys.2026.1802999

**Published:** 2026-04-13

**Authors:** Dandan Li, Yang He, Zhiqiang Fan, Zhen Chen, Guiming Deng, Linqi Ouyang

**Affiliations:** 1 Department of Discipline Construction and Scientific Research Management, The First Hospital of Hunan University of Chinese Medicine, Changsha, China; 2 Department of Pharmacy, The First Hospital of Hunan University of Chinese Medicine, Changsha, China

**Keywords:** gabapentinoids, gabapentin, pregabalin, combination therapy, monotherapy, neuropathic pain

## Abstract

**Background:**

Whether combining gabapentinoids with other agents yields superior efficacy and safety outcomes compared to gabapentinoid monotherapy in patients with neuropathic pain remains unknown.

**Objective:**

To compare the efficacy and safety of gabapentinoid combination therapy versus monotherapy in patients with neuropathic pain in head-to-head comparative studies.

**Methods:**

PubMed, Web of Science, Embase, and Cochrane Library were systematically searched from inception to November 4, 2025. Data abstraction and quality assessment were conducted in accordance with the Preferred Reporting Items for Systematic Reviews and Meta-Analyses (PRISMA) reporting guideline and the Cochrane risk-of-bias tool, respectively. Pain scores (standardized to a 0–10 scale) were the primary outcomes, sleep interference scores, Patient Global Impression of Change (PGIC) and adverse events were the secondary outcomes. Study screening and selection were performed independently by 2 reviewers, with any disagreements resolved by a third adjudicator. Heterogeneity among studies was assessed using the *I*^2^ statistic.

**Results:**

Twenty-one clinical trials comprising 2204 patients were included in this meta-analysis. Gabapentinoid combination therapy was superior to monotherapy in reducing pain (MD = − 1.27, 95% CI = − 1.55 to − 0.99; n =18) and sleep interference scores (MD = − 0.92, 95% CI = − 1.40 to − 0.45; n =5) and increasing the PGIC response rate (RR = 1.80, 95% CI = 1.36 to 2.39; n =4). Subgroup analyses demonstrated that gabapentinoids, both gabapentin and pregabalin, achieved statistically significant greater pain reduction when combined with other gabapentinoids or antidepressants, dietary supplements, local anesthetic, non-pharmacological treatment, and opioids, whereas only a non-significant decreasing trend with immunomodulators. Notably, patients with painful diabetic neuropathy (PDN) and postherpetic neuralgia (PHN) may benefit more from combination therapy. Although combination therapy was associated with higher overall discontinuation rates and certain adverse events, these safety concerns were largely driven by opioid–gabapentinoid combinations; most other combinations had a safety profile comparable to monotherapy.

**Conclusion:**

Gabapentinoid combination therapy was more effective than monotherapy for neuropathic pain, but the benefit-risk profile of specific combinations warrants careful consideration in clinical decision-making.

**Systematic review registration:**

https://www.crd.york.ac.uk/prospero/, identifier CRD420251275655.

## 1 Introduction

Neuropathic pain, defined as pain caused by a lesion or disease of the somatosensory nervous system (International Association for the Study of Pain), is a particularly severe form of chronic pain and significantly undermines patients’ quality of life. The overall prevalence of neuropathic pain was about 9% (Baskozos et al., 2023). Common causes of neuropathic pain include diabetic neuropathy (DN), postherpetic neuralgia (PHN), trigeminal neuralgia (TN), HIV, stroke, painful radiculopathy, and peripheral nerve injury pain (Colloca et al., 2017). The substantial bio-psycho-social burden of neuropathic pain validates chronic pain’s status as a leading cause of worldwide disease burden (Blyth and Huckel Schneider, 2018).

The first-line pharmacological treatments for neuropathic pain are tricyclic antidepressants (TCAs), gabapentinoids (e.g., gabapentin and pregabalin), and serotonin noradrenaline reuptake inhibitors (SNRIs), as strongly recommended by the 2015 guidelines from the Neuropathic Pain Special Interest Group (NeuPSIG) (Finnerup et al., 2015), which were updated and largely confirmed by the latest 2025 systematic review and meta-analysis (Soliman et al., 2025). However, patients often find it difficult to achieve adequate pain relief through standard treatments. Insufficient therapeutic effects and dose-limiting side effects are the main reasons. Notably, approximately 50% of patients do not respond to monotherapy (achieving less than 50% pain reduction from baseline), highlighting the urgent need for more effective therapeutic strategies (Dworkin et al., 2007).

Considering the complexity and heterogeneity of the mechanisms underlying neuropathic pain, combination therapy, which engages multiple complementary pain-relieving pathways, might be a promising strategy to achieve superior efficacy. For patients with painful diabetic neuropathy (PDN), combining gabapentinoids with TCAs or SNRIs provides better pain reduction than monotherapy (Medeiros Dantas et al., 2023). However, other studies have not found evidence that combination therapy (e.g., opioid-antidepressant, opioid-gabapentinoid, or gabapentinoid-antidepressant) is superior to monotherapy in the management of neuropathic pain (Balanaser et al., 2023; Nalamachu et al., 2025). Hence, the efficacy and optimal application of combination therapy, especially how and when to use it, remain undetermined (Claudia, 2025).

Given the lack of conclusive evidence on the efficacy of combination therapy, particularly when it includes the first-line drug gabapentinoids, we conducted a systematic meta-analysis to compare the efficacy and safety of gabapentinoid combination therapy versus monotherapy in patients with neuropathic pain. In contrast to prior meta-analyses of combination therapy for neuropathic pain, our analysis provides novel insights by focusing exclusively on gabapentinoid-based combinations and employing a head-to-head design to better estimate the specific contribution of gabapentinoid combination therapy.

## 2 Methods

### 2.1 Search strategy

We systematically searched the PubMed, Web of Science, Embase, and Cochrane Library from inception to November 4, 2025. The search strategy adhered to PRISMA guidelines and included keywords related to neuropathic pain, PDN, PHN, gabapentinoids, pregabalin, gabapentin and combination therapy. The language was limited to English. Full details of the search strategy are provided in the **Supplementary Materials** in **Appendix S1**. This meta-analysis was registered on PROSPERO, CRD420251275655.

### 2.2 Study selection criteria

Duplicates were removed before study selection using EndNote (version 21) software, initially automatically and then manually verified by the reviewers (D.L. and Y.H.). Study selection of eligible studies was performed by two independent reviewers (D.L. and Y.H.), with discrepancies resolved by discussion. Studies were screened based on the following inclusion criteria: (1) participants are patients with neuropathic pain (e.g., PDN, PHN, TN, peripheral nerve injury, central post stroke pain, multiple sclerosis, phantom limb pain, radiculopathy, or spinal cord injury); (2) comparator and intervention groups consisting of gabapentinoid (e.g., pregabalin, gabapentin, or mirogabalin) monotherapy and combination therapy, respectively; (3) outcomes including pain, sleep interference scores, Patient Global Impression of Change (PGIC), or adverse events; (4) clinical trials. Exclusion criteria were (1) nonhuman studies, (2) gray literature, conference abstracts, letters, case reports, and systematic reviews, (3) clinical trials without outcomes of interest, and (4) cross-over studies without phase-by-phase data, especially the first phase.

### 2.3 Data extraction

Data extraction was performed independently by two reviewers (D.L. and Y.H.), and disagreements were resolved by a third reviewer (Z.F.). Extracted data included study characteristics (first author, year, countries, trial design, treatment duration), participant characteristics (sample size, type of neuropathic pain, age, male sex), outcomes of interest (pain, sleep interference scores, PGIC, adverse events). The primary outcomes were pain scores, standardized to a 0–10 scale (using NRS, VAS, or other validated scales). Sleep interference scores, PGIC and adverse events were the secondary outcomes. Outcome data was extracted at the end of the treatment phase.

### 2.4 Study quality assessment and risk of bias assessment

The Cochrane Collaboration Risk Assessment Tool was used to assess the methodological quality and risk of bias of included studies. Two reviewers (D.L. and Y.H.) independently indicated “low risk of bias”, “high risk of bias” or “unclear risk of bias” to each of the following parameters: random sequence generation, allocation concealment, blinding of participants and personnel, blinding of outcome assessment, incomplete outcome data, selective reporting, and other sources of bias. In addition, we assessed the certainty of evidence for main outcomes using the GRADE approach. Conflicts between reviewers were adjudicated by a third reviewer (Z.F.).

### 2.5 Statistical analyses

Mean difference with 95% confidence interval (CI) were used for continuous variables, while risk ratio (RR) with 95% CI were used for dichotomous variables. In case where mean and Standard Deviation (SD) were not available, we calculated from Standard Error (SE), sample size, *P*-value, first (Q1) and third (Q3) quartiles according to the methodology from the Cochrane Library Handbook. Heterogeneity across studies was assessed using the *I^2^* statistic, with the *I^2^* value of 25%, 50%, and 75% indicating low, moderate, and high heterogeneity, respectively. A random-effects model was utilized if *I^2^* > 50%, otherwise, a fixed-effects model was applied. To assess the stability of gabapentinoid combination therapy versus monotherapy on efficacy outcomes and explore heterogeneity, we performed subgroup analyses by monotherapy agent, class of combination therapy, type of neuropathic pain, and study design. Publication bias was assessed using funnel plot with Egger’s and Begg’s test. Sensitivity analyses were performed by leave-one-out method to assess the robustness of our findings. All analyses were performed using the statistical software Stata 18.0 and Review manager 5.4, *P* < 0.05 was considered statistically significant.

## 3 Results

### 3.1 Study characteristics

**Figure 1:**
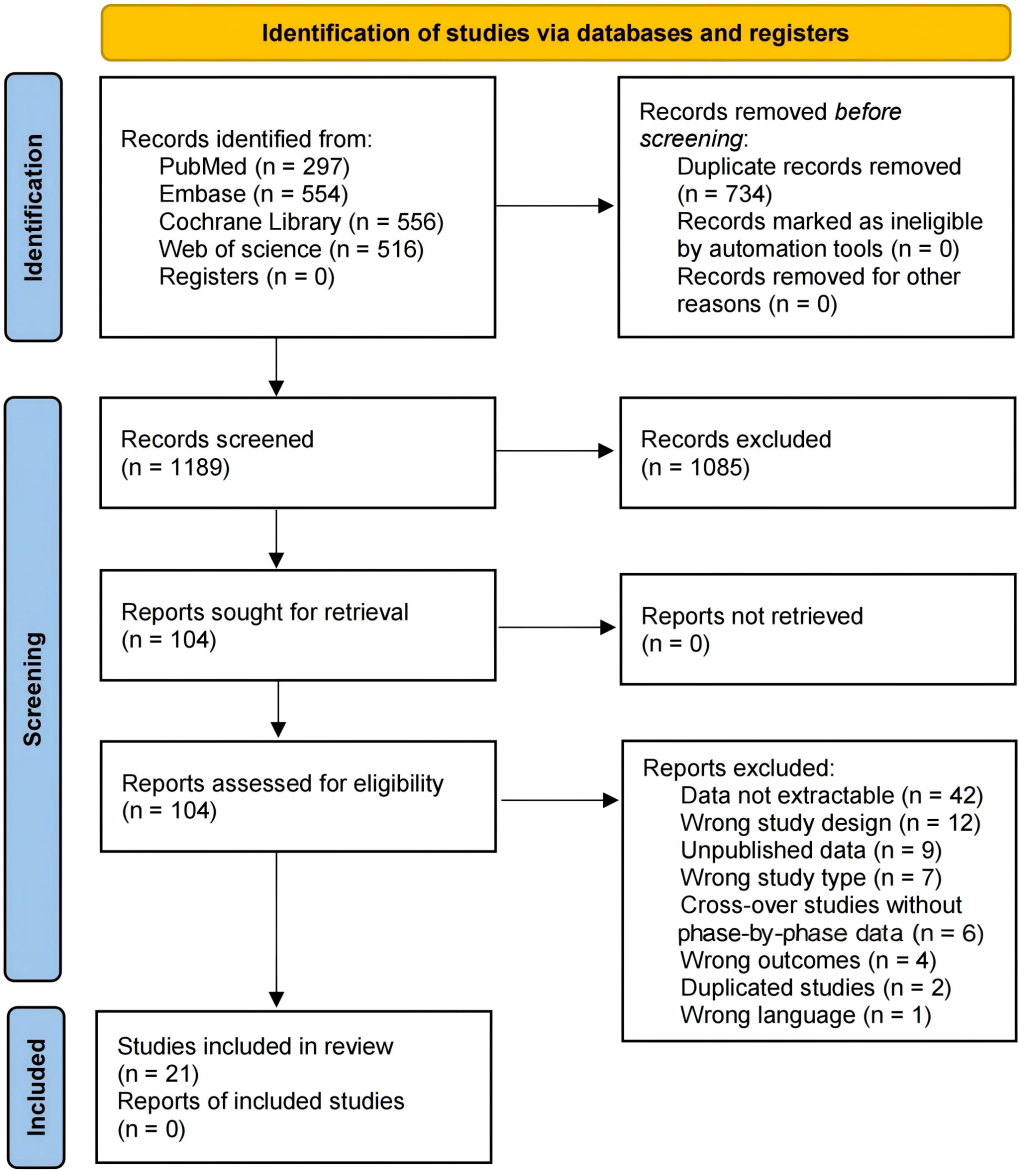
PRISMA flow diagram of studies.

The literature search identified 1923 articles, of which full texts of 104 articles were assessed for eligibility. A total of 21 studies with 2204 participants met the inclusion criteria, published between 2001 and 2025. Details of the study selection are shown in **Figure 1**, and characteristics of the included studies are summarized in **Table 1**. Among the 21 included studies, 10 studies focused on gabapentin and 11 studies focused on pregabalin, all evaluating both monotherapy and combination therapy for patients with neuropathic pain. In all combination therapy, which used gabapentinoids as the basic treatment, the additional treatments were antidepressants (5 trials) (Simpson, 2001; Amr, 2010; Chakrabarty et al., 2019; Saxena et al., 2024; Tesfaye et al., 2024), opioids (4 trials) (Hanna et al., 2008; Gatti et al., 2009; Wang et al., 2017; Huang et al., 2021), dietary supplements (5 trials) (Vasudevan et al., 2014; Altiparmak et al., 2019; Heidari et al., 2019; Shokri et al., 2021; Amini et al., 2022), local anesthetic (1 trial) (Lemos et al., 2008), gabapentinoids (1 trial) (Tao et al., 2025), immunomodulators (1 trial) (Wu et al., 2025), and non-pharmacological treatment (4 trials) (Barbarisi et al., 2010; Huang et al., 2018; Saxena et al., 2021; Yang et al., 2025). Most of the trials were conducted in Asia and Europe. Treatment duration among the 21 included trials ranged from 1 to 12 weeks: six trials were short-term (≤4 weeks), eight trials had an 8-week duration, and seven trials lasted 12 weeks.

**Table 1:** Characteristics of the included studies.

Study ID	Monotherapy	Combination therapy	Combination class	Disease	Treatment period	Dosage		Participants (n)		Age, mean (SD or range), y		Sex (Male), No. (%)	
						Treatment	Control	Treatment	Control	Treatment	Control	Treatment	Control
(Amr, 2010)	Gabapentin	Ketamine + Gabapentin	Antidepressants	SCI	1 w	80 mg/d Ketamine + 900 mg/d Gabapentin	900 mg/d Gabapentin	20	20	48.6 (10.1)	48.7 (9.7)	16 (80.0)	17 (85.0)
(Chakrabarty et al., 2019)	Pregabalin	Amitriptyline + Pregabalin	Antidepressants	Mixed	12 w	10 mg/d Amitriptyline + 75 mg/d Pregabalin	150 mg/d Pregabalin	34	42	/	/	/	/
(Saxena et al., 2024)	Pregabalin	Duloxetine + Pregabalin	Antidepressants	PDN	4 w	60 mg/d Duloxetine + 150 mg/d Pregabalin	150 mg/d Pregabalin	17	17	55.7 (10.0)	53.5 (11.6)	/	/
(Simpson, 2001)	Gabapentin	Venlafaxine + Gabapentin	Antidepressants	PDN	8 w	37.5–150 mg/d Venlafaxine + 300–3600 mg/d Gabapentin	300–3600 mg/d Gabapentin	6	5	/	/	/	/
(Tesfaye et al., 2024)	Gabapentin	Trazodone + Gabapentin	Antidepressants	PDN	8 w	30 mg/d Trazodone + 300 mg/d Gabapentin	≤1800 mg/d Gabapentin	37	43	61.6 (9.2)	63.7 (8.4)	18 (48.6)	22 (51.2)
(Gatti et al., 2009)	Pregabalin	CR Oxycodone + Pregabalin	Opioids	Mixed	90 d	≥ 19.4 mg/d CR Oxycodone + ≥ 108.1 mg/d Pregabalin	≥ 85.6 mg/d Pregabalin	169	134	62 (21-84)	61 (34-77)	76 (45.0)	61 (45.5)
(Hanna et al., 2008)	Gabapentin	PR Oxycodone + Gabapentin	Opioids	PDN	12 w	≥ 10 mg/d PR Oxycodone + ≤ 18000 mg/d Gabapentin	≤ 4800 mg/d Gabapentin	163	165	59.6 (10.5)	60.7 (9.9)	100 (61.3)	110 (66.7)
(Huang et al., 2021)	Pregabalin	Hydromorphone + Pregabalin	Opioids	PHN	12 w	2 mg/d Hydromorphone + 150–300 mg/d Pregabalin	150–300 mg/d Pregabalin	96	97	67.2 (9.6)	66.6 (14.9)	46 (47.9)	51 (52.6)
(Wang et al., 2017)	Pregabalin	Morphine + Pregabalin	Opioids	Mixed	90 d	25.5 mg/d Morphine + 106.3 mg/d Pregabalin (initial dosage)	86.2 mg/d Pregabalin (initial dosage)	128	102	/	/	60 (46.9)	59 (57.8)
(Altiparmak et al., 2019)	Gabapentin	Melatonin + Gabapentin	Dietary supplements	Mixed	30 d	3 mg/d Melatonin + 300–900 mg/d Pregabalin	300–900 mg/d Gabapentin	40	40	49.3 (13.7)	47.2 (12.8)	22 (55.0)	19 (47.5)
(Amini et al., 2022)	Pregabalin	Coenzyme Q10 + Pregabalin	Dietary supplements	PDN	8 w	300 mg/d Coenzyme Q10 + 150 mg/d Pregabalin	150 mg/d Pregabalin	57	55	58.6 (7.7)	59.9 (8.0)	23 (40.4)	21 (38.2)
(Heidari et al., 2019)	Pregabalin	NAC + Pregabalin	Dietary supplements	PDN	8 w	1200 mg/d NAC + 150 mg/d Pregabalin	150 mg/d Pregabalin	43	47	58.8 (6.5)	57.5 (9.0)	7 (16.3)	9 (19.1)
(Shokri et al., 2021)	Pregabalin	Melatonin + Pregabalin	Dietary supplements	PDN	8 w	3–6 mg/d Melatonin + 150 mg/d Pregabalin	150 mg/d Pregabalin	52	51	60.6 (7.2)	58.5 (8.4)	17 (32.7)	15 (29.4)
(Vasudevan et al., 2014)	Pregabalin	Methylcobalamin + ALA +Pregabalin	Dietary supplements	PDN	12 w	1.5 mg/d Methylcobalamin + 200 mg/d ALA +150 mg/d Pregabalin	150 mg/d Pregabalin	14	15	56.9 (12.7)	60.7 (7.4)	6 (42.9)	8 (53.3)
(Lemos et al., 2008)	Gabapentin	Ropivacain + Gabapentin	Local anesthetic	TN	4 w	4 mg/w Ropivacain + 100–900 mg/d Gabapentin	100–900 mg/d Gabapentin	12	12	64 (19.2)	61 (10.8)	3 (25.0)	7 (58.3)
(Tao et al., 2025)	Gabapentin	Pregabalin + Gabapentin	Gabapentinoids	PHN	8 w	25–75 mg/d Pregabalin + 300–900 mg/d Gabapentin	300–900 mg/d Gabapentin	67	67	/	/	31 (46.3)	30 (44.8)
(Wu et al., 2025)	Gabapentin	BCG-PSN + Gabapentin	Immunomodulators	PHN	8 w	1 mL BCG-PSN every other day + 300–900 mg/d Gabapentin	300–900 mg/d Gabapentin	49	49	/	/	24 (49.0)	23 (46.9)
(Barbarisi et al., 2010)	Pregabalin	TENS + Pregabalin	Non-pharmacological Treatment	PHN	4 w	TENS + 150–600 mg/d Pregabalin	150–600 mg/d Pregabalin	9	8	/	/	/	/
(Huang et al., 2018)	Gabapentin	Radiofrequency + Gabapentin	Non-pharmacological Treatment	PHN	4 w	Radiofrequency + 2400 mg/d Gabapentin	2400 mg/d Gabapentin	58	58	48.5 (14.6)	46.7 (12.3)	32 (55.2)	29 (50.0)
(Saxena et al., 2021)	Pregabalin	CBT + Pregabalin	Non-pharmacological Treatment	PHN	12 w	CBT + 150 mg/d Pregabalin	150 mg/d Pregabalin	20	20	54.5 (13.5)	57.0 (11.2)	10 (50.0)	12 (60.0)
(Yang et al., 2025)	Gabapentin	Shugan Tiaoshen needling + Gabapentin	Non-pharmacological Treatment	PHN	8 w	Shugan Tiaoshen needling + 300–2400 mg/d Gabapentin	300–2400 mg/d Gabapentin	33	33	58.3 (11.7)	55.9 (11.2)	13 (39.4)	15 (45.5)

SCI, spinal cord injury; PDN, painful diabetic neuropathy; PHN, postherpetic neuralgia; TN, trigeminal neuralgia; CR Oxycodone, controlled-release oxycodone; PR Oxycodone, prolonged-release oxycodone; NAC, N-acetylcysteine; ALA, alpha lipoic acid; BCG-PSN, Bacillus Calmette-Guerin polysaccharide and nucleic acid injection; TENS, transcutaneous electric nerve stimulation; CBT, cognitive behavioral therapy.

### 3.2 Pooled analyses of efficacy outcomes

**Figure 2:**
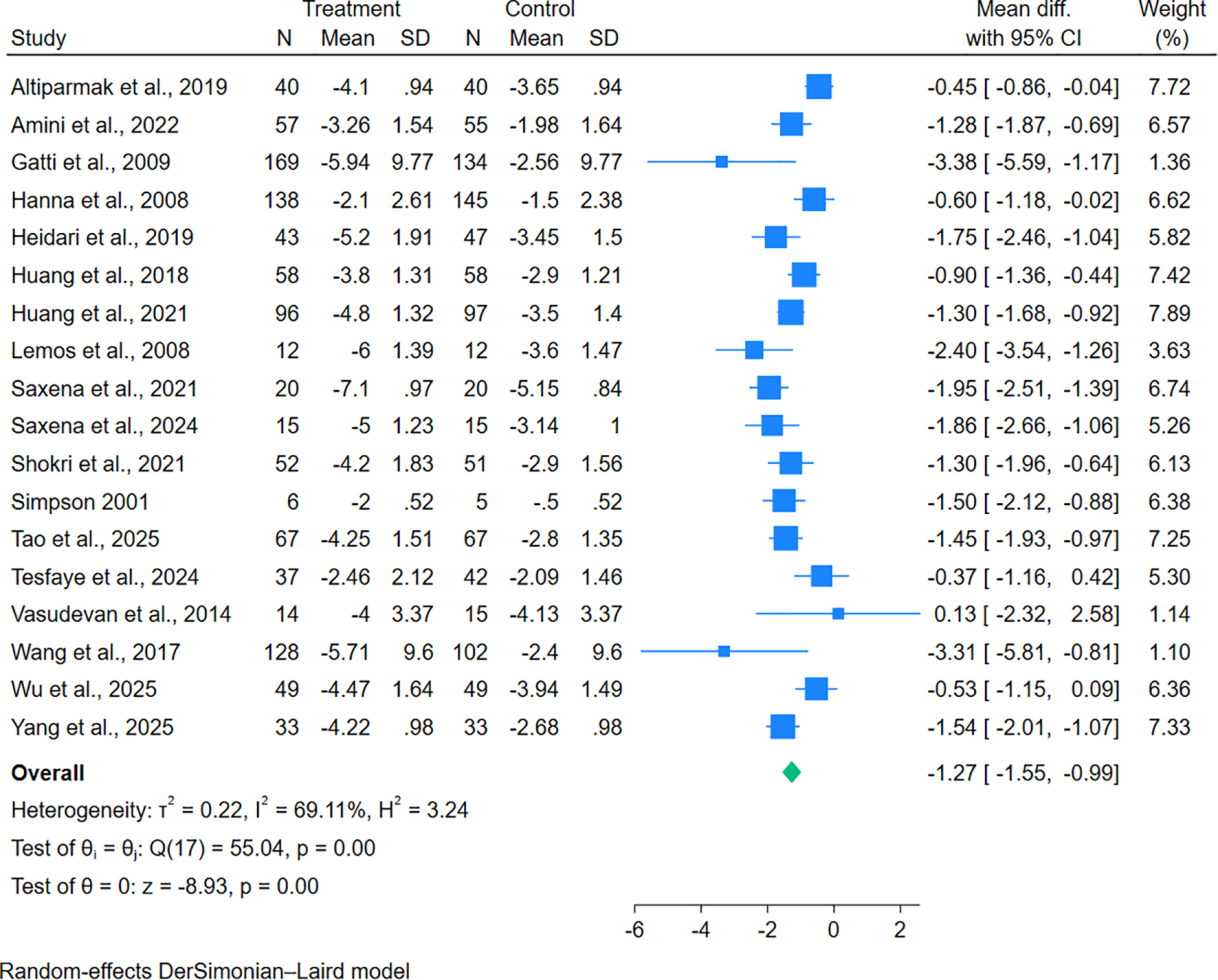
Effects of gabapentinoid combination therapy versus monotherapy on the change of average pain scores from baseline in patients with neuropathic pain.

**Figure 3:**
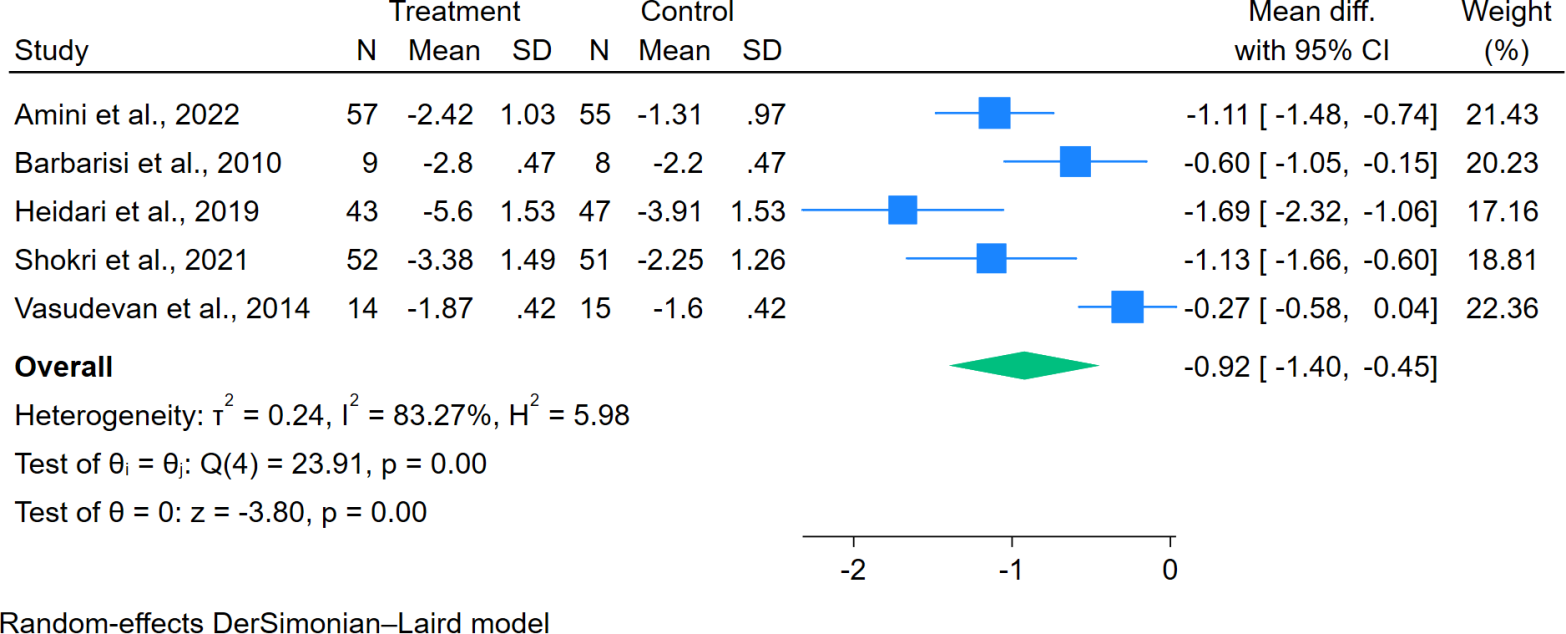
Effects of gabapentinoid combination therapy versus monotherapy on sleep interference scores in patients with neuropathic pain.

**Figure 4:**
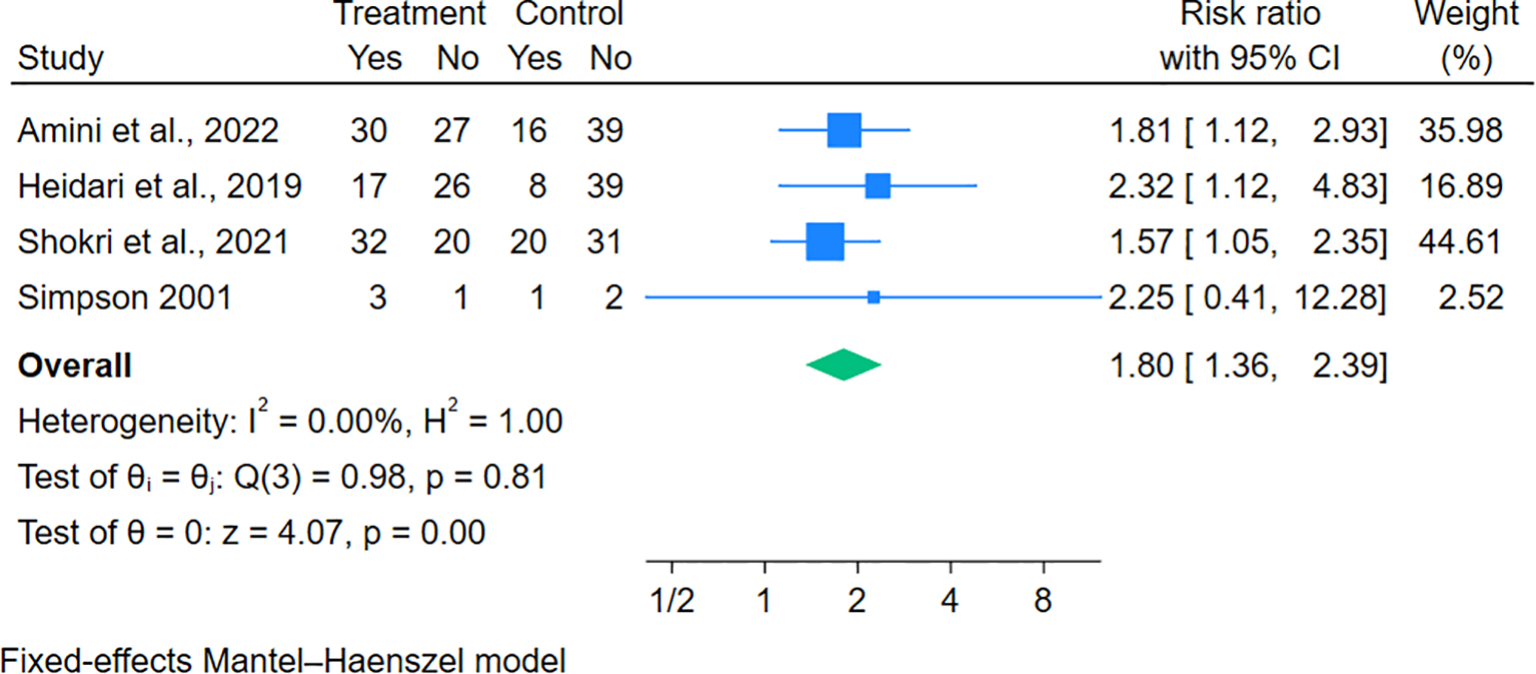
Effects of gabapentinoid combination therapy versus monotherapy on Patient Global Impression of Change (PGIC) in patients with neuropathic pain.

Of 21 studies included, 18 trials provided data on the change of mean pain scores from baseline. Gabapentinoid combination therapy was associated with superior pain reduction relative to monotherapy, with moderate heterogeneity across studies (MD = − 1.27, 95% CI = − 1.55 to − 0.99; n = 18) (**Figure 2**). Similarly, a reduction in sleep interference scores was also significantly reduced in combination therapy compared with monotherapy (MD = − 0.92, 95% CI = − 1.40 to − 0.45; n = 5) (**Figure 3**). In addition, there was a significant increase in the percentage of subjects with PGIC of “very much” or “much improved” in combination therapy compared with monotherapy, with no heterogeneity across studies (RR = 1.80, 95% CI = 1.36 to 2.39; n = 4) (**Figure 4**).

### 3.3 Subgroup analyses of efficacy outcomes

In the subgroup analyses, combining gabapentinoids with antidepressants (MD = − 1.25, 95% CI = − 2.08 to − 0.43; n = 3), dietary supplements (MD = − 1.09, 95% CI = − 1.67 to − 0.52; n =5), gabapentinoids (MD = − 1.45, 95% CI = − 1.93 to − 0.97; n = 1), local anesthetic (MD = − 2.40, 95% CI = − 3.54 to − 1.26; n = 1), non-pharmacological treatment (MD = − 1.45, 95% CI = − 2.04 to − 0.85; n = 3), and opioids (MD = − 1.47, 95% CI = − 2.33 to − 0.60; n = 4) were significantly more efficacious than monotherapy in pain reduction, whereas the combination of gabapentinoids with immunomodulators demonstrated only a non-significant decreasing trend (MD = − 0.53, 95% CI = − 1.15 to 0.09; n = 1) (**Supplementary Figure 1A**). In addition, the combination of gabapentinoids with dietary supplements can significantly reduce the sleep interference scores (MD = − 1.01, 95% CI = − 1.62 to – 0.41; n = 4) (**Supplementary Figure 2A**) and improve the percentage of subjects with PGIC of “very much” or “much improved” (RR = 1.79, 95% CI = 1.34 to 2.38; n = 3) (**Supplementary Figure 3A**).

Both gabapentin (MD = − 1.03, 95% CI = − 1.40 to – 0.66; n = 9) and pregabalin (MD = − 1.57, 95% CI = − 1.89 to – 1.26; n = 9) were more effective in alleviating pain when combined with other agents than as monotherapy (**Supplementary Figure 1B**). Additionally, pregabalin combination therapy can significantly decrease sleep interference scores (MD = − 0.92, 95% CI = − 1.40 to − 0.45; n = 5) (**Supplementary Figure 2B**) and improve the PGIC response rate (RR = 1.79, 95% CI = 1.34 to 2.38; n = 3) (**Supplementary Figure 3B**) compared with monotherapy. However, with limited data, no evidence was found that gabapentin combination therapy is more effective than monotherapy in decreasing sleep interference scores and improving the PGIC response rate (**Supplementary Figure 3B**).

Subgroup analyses based on the type of neuropathic pain indicated that gabapentinoid combination therapy can significantly reduce pain compared to monotherapy in patients with PDN (MD = − 1.20, 95% CI = − 1.59 to – 0.81; n = 8), PHN (MD = − 1.29, 95% CI = − 1.64 to – 0.94; n = 6) and TN (MD = − 2.40, 95% CI = − 3.54 to – 1.26; n = 1), whereas only a non-significant trend toward reduction was observed in patients with mixed neuropathic pain (MD = − 2.15, 95% CI = − 4.49 to 0.19; n = 3) (**Supplementary Figure 1C**). In addition, compared to monotherapy, gabapentinoid combination therapy demonstrated a significant reduction in sleep interference scores and an increase in PGIC response rate in patients with PDN and (or) PHN (**Supplementary Figures 2C**, **3C**).

Moreover, in order to evaluate the potential influence of study design, subgroup analyses based on blinding and randomization status were conducted specifically for the outcomes of pain. The subgroup analyses showed that gabapentinoid combination therapy could significantly reduce pain in all study types, suggesting that the overall conclusion is not influenced by the specific study design (**Supplementary Figure 1D**). However, it is noteworthy that the open-label, non-randomized trial had a markedly greater pain reduction than the blinded/randomized trials (**Supplementary Figure 1D**).

### 3.4 Treatment discontinuation and adverse events

Discontinuation of therapy due to adverse events was significantly higher in combination therapy compared with monotherapy (RR = 1.71, 95% CI = 1.14 to 2.57; n = 8) (**Supplementary Figure 4**). Safety results for the most common adverse events are summarized in **Table 2**, and further details are provided in the **Supplementary Materials**. Compared with monotherapy, gabapentinoid combination therapy was associated with significantly higher risk of dizziness (RR = 1.48, 95% CI = 1.07 to 2.06; n = 10) (**Supplementary Figure 5**), somnolence (RR = 1.89, 95% CI = 1.30 to 2.76; n = 7) (**Supplementary Figure 6**), nausea (RR = 2.00, 95% CI = 1.35 to 2.97; n = 7) (**Supplementary Figure 7**), and fatigue (RR = 2.10, 95% CI = 1.23 to 3.57; n = 3) (**Supplementary Figure 8**), while no significant difference was found with constipation (RR = 1.29, 95% CI = 0.24 to 6.94; n = 3) (**Supplementary Figure 9**), headache (RR = 1.30, 95% CI = 0.77 to 2.20; n = 5) (**Supplementary Figure 10**), diarrhea (RR = 0.65, 95% CI = 0.19 to 2.19; n = 3) (**Supplementary Figure 11**), and vomiting (RR = 2.12, 95% CI = 0.98 to 4.59; n = 3) (**Supplementary Figure 12**). Notably, subgroup analyses indicated that, compared with monotherapy, the combination of gabapentinoids and opioids led to a significantly higher risk of both adverse events and discontinuations due to adverse events, whereas the combination of gabapentinoids with other treatments showed no significant difference (**Supplementary Figures 4**-**9**).

**Table 2:** Summary of the most common adverse events.

Adverse event	No. studies	No. events	Sample size	RR (95% CI)	*I*² %	*P*_Heterogeneity_	*P*_Overall effect_
Dizziness	10	129	1007	1.48 (1.07, 2.06)	16.01	0.30	0.02
Nausea	7	94	725	2.00 (1.35, 2.97)	0.00	0.58	< 0.01
Somnolence	7	99	702	1.89 (1.30, 2.76)	46.99	0.08	< 0.01
Constipation	3	66	550	1.29 (0.24, 6.94)	76.90	0.01	0.77
Headache	5	51	579	1.30 (0.77, 2.20)	0.00	0.57	0.32
Fatigue	3	56	572	2.10 (1.23, 3.57)	0.00	0.78	0.01
Vomiting	3	28	459	2.12 (0.98, 4.59)	0.00	0.85	0.06
Diarrhea	3	10	203	0.65 (0.19, 2.19)	0.00	0.85	0.49

### 3.5 Quality assessment

**Figure 5:**
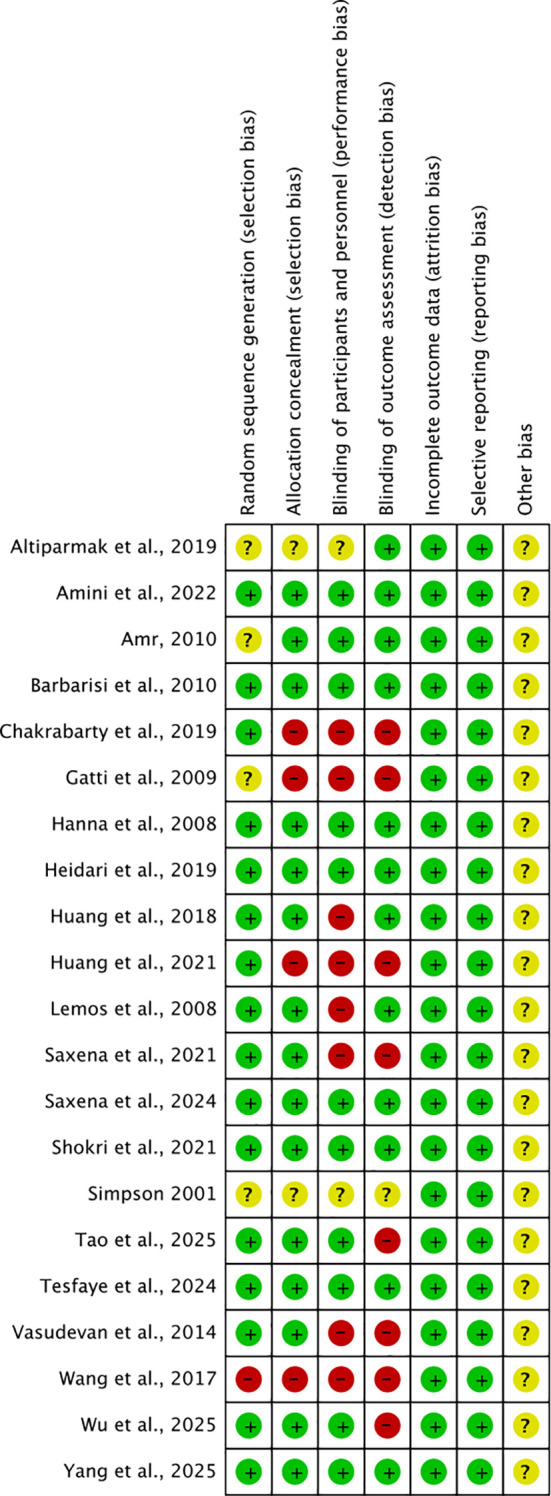
Risk of bias summary. Green: low risk of bias; yellow : unclear risk of bias; red: high risk of bias.

The methodological quality of included studies was assessed by the Cochrane Collaboration Risk Assessment Tool (**Figure 5**). Among the 21 studies included, 11 were double-blind studies, 3 were single-blind studies, and 7 were open-label studies. Sixteen and fifteen studies were classified as having a “low risk” of random sequence generation and allocation concealment (selection bias), respectively. In performance bias, 11 studies were rated as “low risk” of bias, 8 as “high risk”, and 2 as “unclear”. For detection bias, the majority (n=12) were judged to have a “low risk”, while 8 had a “high risk”, and 1 had an “unclear risk”. All studies were rated as having “unclear risk” of other bias, and none of the included studies had attrition bias and reporting bias. Additionally, GRADE assessment demonstrated moderate-certainty evidence for the effects of combination therapy on pain and sleep interference scores, and high-certainty evidence for PGIC (**Table 3**).

**Table 3:** GRADE assessment of included studies on main comparison.

Certainty assessment							№ of patients		Effect		Certainty	Importance
№ of studies	Study design	Risk of bias	Inconsistency	Indirectness	Imprecision	Other considerations	gabapentinoid combination therapy	monotherapy	Relative (95% CI)	Absolute (95% CI)		
Pain scores												
18	randomized trials	not serious	serious^a^	not serious	not serious	none	1037	987	–	MD 1.27 lower (1.55 lower to 0.99 lower)	⊕⊕⊕◯ Moderate^a^	CRITICAL
Sleep interference scores												
5	randomized trials	not serious	serious^a^	not serious	not serious	none	175	176	–	MD 0.92 lower (1.4 lower to 0.45 lower)	⊕⊕⊕◯ Moderate^a^	CRITICAL
Patient Global Impression of Change (PGIC)												
4	randomized trials	not serious	not serious	not serious	not serious	none	82/156 (52.6%)	45/156 (28.8%)	RR 1.80 (1.36 to 2.39)	231 more per 1,000 (from 104 more to 401 more)	⊕⊕⊕⊕ High	CRITICAL

CI, confidence interval; MD, mean difference; RR, risk ratio.

^a^Downgraded once for inconsistency (high heterogeneity).

### 3.6 Publication bias and sensitivity analyses

Based on the results of Egger’s and Begg’s tests, no publication bias was detected in the primary outcomes of the analyses (**Supplementary Figure 13**). The leave-one-out sensitivity analyses were conducted to evaluate the robustness of the primary findings. The efficacy outcomes of pain, sleep interference scores, and PGIC remained consistent with the full-sample results after excluding any individual trial, supporting the reliability of the main findings (**Supplementary Figure 14**).

## 4 Discussion

Our meta-analysis demonstrated that gabapentinoid combination therapy provides superior efficacy in reducing pain and sleep interference scores, and increasing the PGIC response rate compared with monotherapy, particularly in patients with PDN and PHN. Overall, combination therapy was associated with higher rates of common adverse events (e.g., dizziness, somnolence, nausea, and fatigue) and treatment discontinuation compared to monotherapy. Notably, this increase was largely attributable to opioid–gabapentinoid combinations; most non-opioid regimens demonstrated a tolerability profile comparable to monotherapy. Thus, combination therapy may be a potentially promising strategy for neuropathic pain. However, these findings were based on short-term trials (1–12 weeks) with considerable heterogeneity across studies, and long-term data remain limited. Further large-scale, long-term studies are needed to confirm sustained efficacy and safety.

Our findings suggested that combining gabapentinoids with other gabapentinoids or antidepressants, dietary supplements, local anesthetic, non-pharmacological treatment, and opioids were more effective in the treatment of neuropathic pain compared with monotherapy, while no significant difference was found when combined with immunomodulators. The evidence supporting the superior efficacy of the combination of two gabapentinoids (pregabalin-gabapentin) and the local anesthetic-gabapentinoid combination therapy (ropivacain-gabapentin) was limited, since only one trial for each combination was included in our meta-analysis. To some extent, our data consistent with previous studies, which demonstrated that combination therapy with gabapentinoids, TCAs, or SNRIs may be superior to monotherapy in patients with PDN (Medeiros Dantas et al., 2023). However, other findings suggested that the combination of gabapentinoid-opioid or gabapentinoid-antidepressant do not show superiority over monotherapy in neuropathic pain (Balanaser et al., 2023). The discrepancies may be attributed to different research aims and methodologies: (1) we restricted controls to gabapentinoids, not placebo or other drugs; (2) only clinical trials with identical treatment duration across comparison groups were included in our analyses; (3) we excluded cross-over studies without phase-by-phase data to avoid carryover effects and serious estimation bias. In addition, our analyses included several recently published trials, which provide an updated evidence synthesis. Notably, the pooled mean difference in average pain changed from baseline between combination therapy and monotherapy was small, only 1.27 on a 0–10 scale, which falls below most established minimal clinically important difference (MCID) thresholds for neuropathic pain. Although this estimate was derived from 18 studies and remained robust in leave-one-out sensitivity analyses, this modest effect may not translate into clinically meaningful improvement in real-world patient outcomes.

A notable finding from our analyses is that gabapentinoids were significantly more effective when combined with dietary supplements and non-pharmacological treatment than monotherapy. Five studies included in our meta-analysis assessed the efficacy of combinations with dietary supplements, mainly in patients with PDN, focusing on four specific adding agents, melatonin, Coenzyme Q10, N-acetylcysteine (NAC), methylcobalamin and alpha lipoic acid (ALA). Since inflammation and oxidative stress are key pathological processes in neuropathic pain progression, it has been hypothesized that some dietary supplements may possess anti-inflammatory or antioxidant properties that could complement the analgesic effects of conventional neuropathic pain agents (Grinberg et al., 2005; Cordero et al., 2014; Sandireddy et al., 2014; Vikram et al., 2014; Emet et al., 2016; Sawaddiruk et al., 2019; Nádró et al., 2021). In addition, our analyses included four studies that investigated the combination of gabapentinoids and non-pharmacological treatment, including transcutaneous electric nerve stimulation (TENS), radiofrequency, acupuncture, and cognitive behavioral therapy (CBT). Data from these randomized trials indicated that the combination of gabapentinoids and non-pharmacological treatment was better than monotherapy in patients with PHN, as it led to better pain control and sleep improvement. Mechanistically, gabapentinoids reduce pain primarily by binding to voltage-gated calcium channel α2δ subunits, while non-pharmacological treatment combination allows for complementary mechanisms of action, such as inhibit inflammatory response and nerve impulse conduction (Radhakrishnan and Sluka, 2003; Radhakrishnan et al., 2003; Sluka and Walsh, 2003; Ainsworth et al., 2006; Dworkin et al., 2013). The exact mechanisms underlying combination therapy remain to be elucidated and warrant further investigation. Due to limited number of studies, our subgroup analyses were restricted to treatment categories rather than specific treatment protocols.

For safety analyses, compared with monotherapy, although combination therapy significantly increased the risk of adverse events and discontinuations due to adverse events, subgroup analyses showed that the increased risk mainly comes from the combination therapy of opioid-gabapentinoid. Therefore, it seems safe to combine gabapentinoids with other agents, except for opioids, in the treatment of neuropathic pain. Given that the adverse effects of gabapentinoids and many other agents are predominantly dose-dependent, combination therapy allows the use of lower doses of each drug, which may decrease the frequency of adverse events and lead to a better tolerability profile. Previous studies have shown that adverse events associated with opioids were more persistent, particularly constipation, and the dropout rate due to adverse events with opioids was high in neuropathic pain (Harati et al., 1998; Watson and Babul, 1998; Dworkin et al., 2007). Thus, particular care should be taken when combining gabapentinoids with opioids. Additionally, future studies are warranted to optimize the combination of gabapentinoids with other treatments to improve safety and achieve long-term pain relief.

Our systematic meta-analysis has several strengths. A key strength of the analyses is head-to-head comparisons of combination therapy versus monotherapy with gabapentinoids, providing direct and robust comparative evidence regarding efficacy and safety. In addition, our analyses were restricted to studies with same dosing time among treatment arms, which minimizes the potential confounding impact of dosing time on therapeutic outcomes. Furthermore, the integrated analyses of both pharmacological and non-pharmacological combination therapy offer a uniquely comprehensive evaluation of different treatment strategies, providing evidence for the development of more personalized and effective treatment plans.

There are some methodological limitations to our analyses. First, some of the outcomes showed significant between-trial heterogeneity, which might be attributed to the differences in participant phenotypes, dose titration methods, and trial design. Second, the evidence for certain combinations (e.g., dual gabapentinoids, ropivacaine–gabapentin, BCG-PSN-gabapentin) was based on single small studies, which limits the reliability of subgroup analyses for these specific regimens. In addition, conducting multiple comparisons in subgroup analyses increases the risk of type I error. As such, these findings require validation in future studies. Moreover, several trials included are open-label. However, our subgroup analyses based on the trial type showed that the results were consistent among those trials and double-blinded trials for the primary outcomes.

## 5 Conclusions

In conclusion, our findings demonstrated that gabapentinoid combination therapy was superior to monotherapy in reducing pain and sleep interference scores and increasing the PGIC response rate in patients with neuropathic pain. Although combination therapy overall showed higher rates of adverse events and discontinuations, this excess risk was largely confined to opioid–gabapentinoid combination, while most other regimens exhibited tolerability comparable to monotherapy. Thus, this preliminary evidence indicates that gabapentinoid combination therapy may be a promising alternative for patients with neuropathic pain who do not respond to monotherapy. Further studies are warranted to optimize the combination to achieve long-term efficacy and safety.

## Data Availability

The original contributions presented in the study are included in the article/**Supplementary Material**. Further inquiries can be directed to the corresponding authors.
