# Supplementary material for: Efficacy and safety of gabapentinoid combination therapy versus monotherapy for the treatment of neuropathic pain
Source: Front Physiol. 2026 Apr 13;17:1802999. doi: 10.3389/fphys.2026.1802999 (PMC13110976; doi:10.3389/fphys.2026.1802999)
Supplement: Supplementary file 1 [file DataSheet1.pdf]

Supplementary Material

Supplementary figures and appendix

Figure S1. Subgroup analyses of the effects of gabapentinoid combination therapy versus monotherapy on the change of average pain scores from baseline in patients with neuropathic pain.

Figure S2. Subgroup analyses of the effects of gabapentinoid combination therapy versus monotherapy on sleep interference scores in patients with neuropathic pain.

Figure S3. Subgroup analyses of the effects of gabapentinoid combination therapy versus monotherapy on Patient Global Impression of Change (PGIC) in patients with neuropathic pain.

Figure S4. Risk of discontinuations due to adverse events between gabapentinoid combination therapy versus monotherapy.

Figure S5. Risk of dizziness between gabapentinoid combination therapy versus monotherapy.

Figure S6. Risk of somnolence between gabapentinoid combination therapy versus monotherapy.

Figure S7. Risk of nausea between gabapentinoid combination therapy versus monotherapy.

Figure S8. Risk of fatigue between gabapentinoid combination therapy versus monotherapy.

Figure S9. Risk of constipation between gabapentinoid combination therapy versus monotherapy.

Figure S10. Risk of headache between gabapentinoid combination therapy versus monotherapy.

Figure S11. Risk of diarrhea between gabapentinoid combination therapy versus monotherapy.

Figure S12. Risk of vomiting between gabapentinoid combination therapy versus monotherapy.

Figure S13. Funnel plot for the assessment of publication bias.

Figure S14. Sensitivity analyses by leave-one-out method.

Supplementary Appendix S1: Supplementary Methods.

A

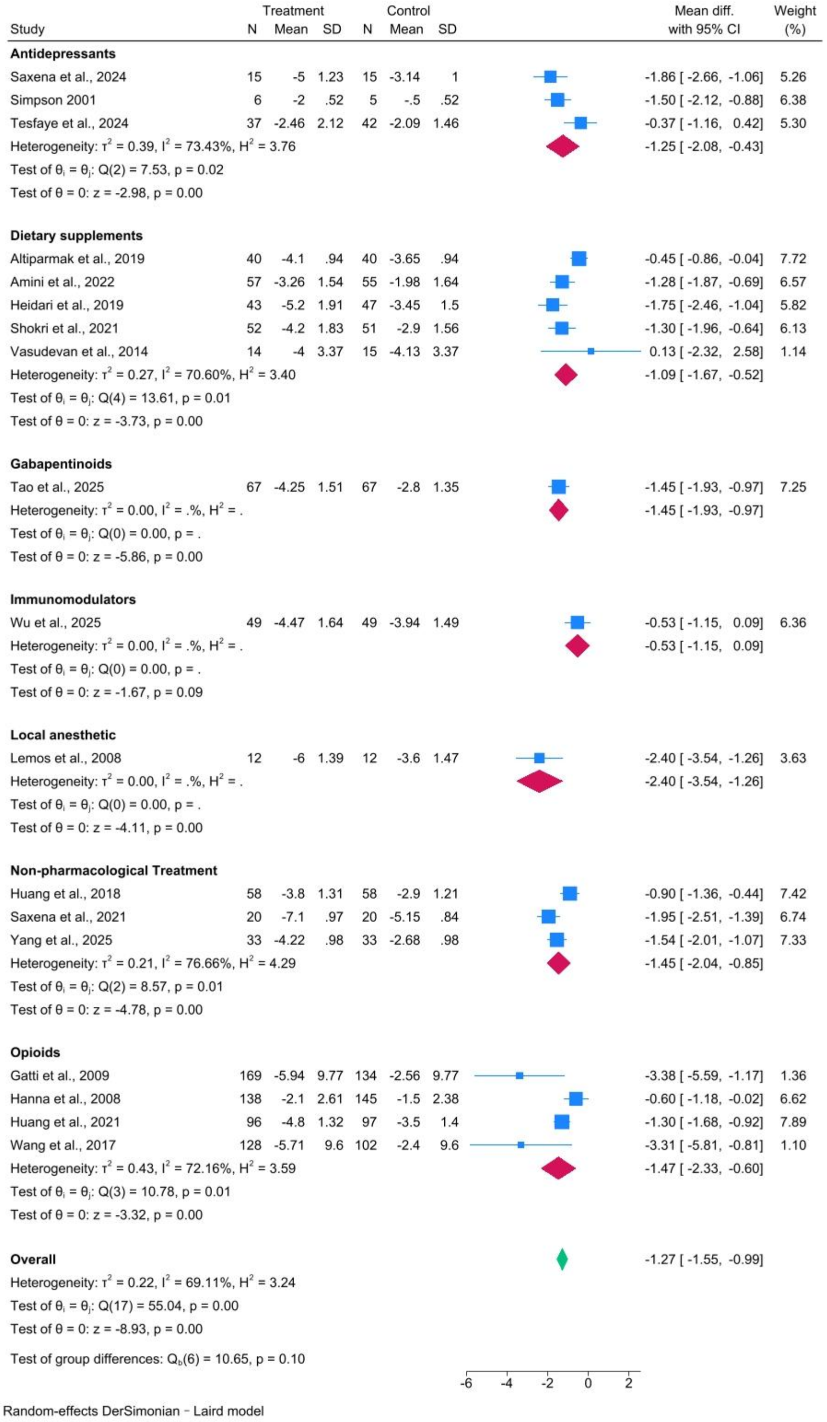
Study
Treatment N Mean SD
Control N Mean SD
Mean diff. with 95% CI
Weight (%)
Antidepressants
Saxena et al., 2024 15 -5 1.23 15 -3.14 1 -1.86 [-2.66, -1.06] 5.26
Simpson 2001 6 -2 .52 5 -.5 .52 -1.50 [-2.12, -0.88] 6.38
Tesfaye et al., 2024 37 -2.46 2.12 42 -2.09 1.46 -0.37 [-1.16, 0.42] 5.30
Heterogeneity: τ2 = 0.39, I2 = 73.43%, H2 = 3.76 -1.25 [-2.08, -0.43]
Test of θi = θj: Q(2) = 7.53, p = 0.02
Test of θ = 0: z = -2.98, p = 0.00
Dietary supplements
Altiparmak et al., 2019 40 -4.1 .94 40 -3.65 .94 -0.45 [-0.86, -0.04] 7.72
Amini et al., 2022 57 -3.26 1.54 55 -1.98 1.64 -1.28 [-1.87, -0.69] 6.57
Heidari et al., 2019 43 -5.2 1.91 47 -3.45 1.5 -1.75 [-2.46, -1.04] 5.82
Shokri et al., 2021 52 -4.2 1.83 51 -2.9 1.56 -1.30 [-1.96, -0.64] 6.13
Vasudevan et al., 2014 14 -4 3.37 15 -4.13 3.37 0.13 [-2.32, 2.58] 1.14
Heterogeneity: τ2 = 0.27, I2 = 70.60%, H2 = 3.40 -1.09 [-1.67, -0.52]
Test of θi = θj: Q(4) = 13.61, p = 0.01
Test of θ = 0: z = -3.73, p = 0.00
Gabapentinoids
Tao et al., 2025 67 -4.25 1.51 67 -2.8 1.35 -1.45 [-1.93, -0.97] 7.25
Heterogeneity: τ2 = 0.00, I2 = .%, H2 = . -1.45 [-1.93, -0.97]
Test of θi = θj: Q(0) = 0.00, p = .
Test of θ = 0: z = -5.86, p = 0.00
Immunomodulators
Wu et al., 2025 49 -4.47 1.64 49 -3.94 1.49 -0.53 [-1.15, 0.09] 6.36
Heterogeneity: τ2 = 0.00, I2 = .%, H2 = . -0.53 [-1.15, 0.09]
Test of θi = θj: Q(0) = 0.00, p = .
Test of θ = 0: z = -1.67, p = 0.09
Local anesthetic
Lemos et al., 2008 12 -6 1.39 12 -3.6 1.47 -2.40 [-3.54, -1.26] 3.63
Heterogeneity: τ2 = 0.00, I2 = .%, H2 = . -2.40 [-3.54, -1.26]
Test of θi = θj: Q(0) = 0.00, p = .
Test of θ = 0: z = -4.11, p = 0.00
Non-pharmacological Treatment
Huang et al., 2018 58 -3.8 1.31 58 -2.9 1.21 -0.90 [-1.36, -0.44] 7.42
Saxena et al., 2021 20 -7.1 .97 20 -5.15 .84 -1.95 [-2.51, -1.39] 6.74
Yang et al., 2025 33 -4.22 .98 33 -2.68 .98 -1.54 [-2.01, -1.07] 7.33
Heterogeneity: τ2 = 0.21, I2 = 76.66%, H2 = 4.29 -1.45 [-2.04, -0.85]
Test of θi = θj: Q(2) = 8.57, p = 0.01
Test of θ = 0: z = -4.78, p = 0.00
Opioids
Gatti et al., 2009 169 -5.94 9.77 134 -2.56 9.77 -3.38 [-5.59, -1.17] 1.36
Hanna et al., 2008 138 -2.1 2.61 145 -1.5 2.38 -0.60 [-1.18, -0.02] 6.62
Huang et al., 2021 96 -4.8 1.32 97 -3.5 1.4 -1.30 [-1.68, -0.92] 7.89
Wang et al., 2017 128 -5.71 9.6 102 -2.4 9.6 -3.31 [-5.81, -0.81] 1.10
Heterogeneity: τ2 = 0.43, I2 = 72.16%, H2 = 3.59 -1.47 [-2.33, -0.60]
Test of θi = θj: Q(3) = 10.78, p = 0.01
Test of θ = 0: z = -3.32, p = 0.00
Overall -1.27 [-1.55, -0.99]
Heterogeneity: τ2 = 0.22, I2 = 69.11%, H2 = 3.24
Test of θi = θj: Q(17) = 55.04, p = 0.00
Test of θ = 0: z = -8.93, p = 0.00
Test of group differences: Qb(6) = 10.65, p = 0.10
-6 -4 -2 0 2
Random-effects DerSimonian – Laird model

B

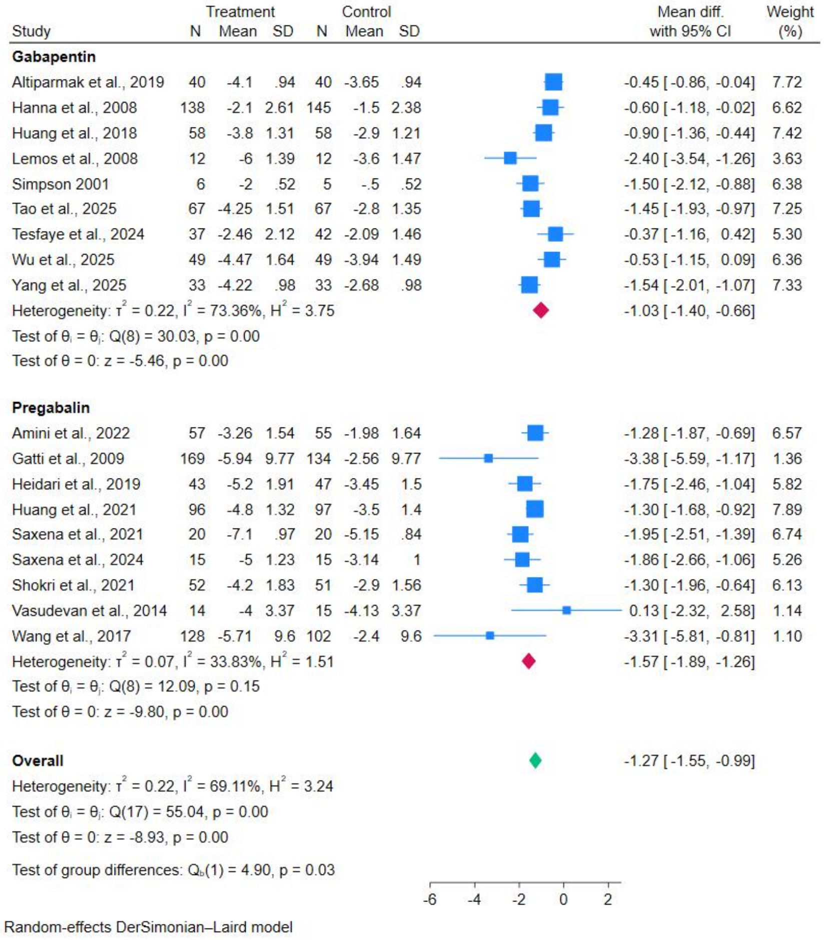

Treatment
Control
Study N Mean SD N Mean SD
Mean diff. with 95% CI
Weight (%)
Gabapentin
Altiparmak et al., 2019 40 -4.1 .94 40 -3.65 .94 -0.45 [-0.86, -0.04] 7.72
Hanna et al., 2008 138 -2.1 2.61 145 -1.5 2.38 -0.60 [-1.18, -0.02] 6.62
Huang et al., 2018 58 -3.8 1.31 58 -2.9 1.21 -0.90 [-1.36, -0.44] 7.42
Lemos et al., 2008 12 -6 1.39 12 -3.6 1.47 -2.40 [-3.54, -1.26] 3.63
Simpson 2001 6 -2 .52 5 -.5 .52 -1.50 [-2.12, -0.88] 6.38
Tao et al., 2025 67 -4.25 1.51 67 -2.8 1.35 -1.45 [-1.93, -0.97] 7.25
Tesfaye et al., 2024 37 -2.46 2.12 42 -2.09 1.46 -0.37 [-1.16, 0.42] 5.30
Wu et al., 2025 49 -4.47 1.64 49 -3.94 1.49 -0.53 [-1.15, 0.09] 6.36
Yang et al., 2025 33 -4.22 .98 33 -2.68 .98 -1.54 [-2.01, -1.07] 7.33
Heterogeneity: τ² = 0.22, I² = 73.36%, H² = 3.75 -1.03 [-1.40, -0.66]
Test of θi = θj: Q(8) = 30.03, p = 0.00
Test of θ = 0: z = -5.46, p = 0.00
Pregabalin
Amini et al., 2022 57 -3.26 1.54 55 -1.98 1.64 -1.28 [-1.87, -0.69] 6.57
Gatti et al., 2009 169 -5.94 9.77 134 -2.56 9.77 -3.38 [-5.59, -1.17] 1.36
Heidari et al., 2019 43 -5.2 1.91 47 -3.45 1.5 -1.75 [-2.46, -1.04] 5.82
Huang et al., 2021 96 -4.8 1.32 97 -3.5 1.4 -1.30 [-1.68, -0.92] 7.89
Saxena et al., 2021 20 -7.1 .97 20 -5.15 .84 -1.95 [-2.51, -1.39] 6.74
Saxena et al., 2024 15 -5 1.23 15 -3.14 1 -1.86 [-2.66, -1.06] 5.26
Shokri et al., 2021 52 -4.2 1.83 51 -2.9 1.56 -1.30 [-1.96, -0.64] 6.13
Vasudevan et al., 2014 14 -4 3.37 15 -4.13 3.37 0.13 [-2.32, 2.58] 1.14
Wang et al., 2017 128 -5.71 9.6 102 -2.4 9.6 -3.31 [-5.81, -0.81] 1.10
Heterogeneity: τ² = 0.07, I² = 33.83%, H² = 1.51 -1.57 [-1.89, -1.26]
Test of θi = θj: Q(8) = 12.09, p = 0.15
Test of θ = 0: z = -9.80, p = 0.00
Overall -1.27 [-1.55, -0.99]
Heterogeneity: τ² = 0.22, I² = 69.11%, H² = 3.24
Test of θi = θj: Q(17) = 55.04, p = 0.00
Test of θ = 0: z = -8.93, p = 0.00
Test of group differences: Qb(1) = 4.90, p = 0.03
-6 -4 -2 0 2
Random-effects DerSimonian–Laird model

C

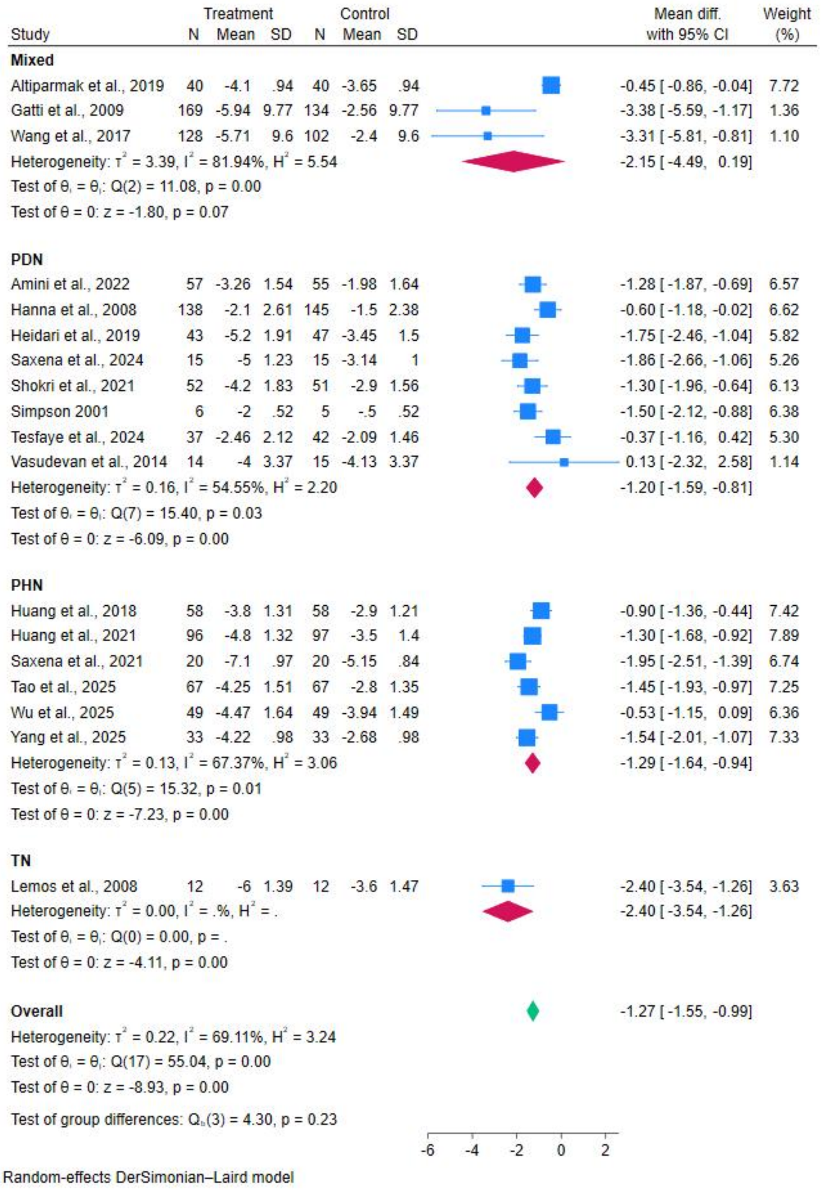
Study
Treatment N Mean SD
Control N Mean SD
Mean diff. with 95% CI
Weight (%)
Mixed
Altiparmak et al., 2019 40 -4.1 .94 40 -3.65 .94 -0.45 [-0.86, -0.04] 7.72
Gatti et al., 2009 169 -5.94 9.77 134 -2.56 9.77 -3.38 [-5.59, -1.17] 1.36
Wang et al., 2017 128 -5.71 9.6 102 -2.4 9.6 -3.31 [-5.81, -0.81] 1.10
Heterogeneity: τ² = 3.39, I² = 81.94%, H² = 5.54 -2.15 [-4.49, 0.19]
Test of θi = θj: Q(2) = 11.08, p = 0.00
Test of θ = 0: z = -1.80, p = 0.07
PDN
Amini et al., 2022 57 -3.26 1.54 55 -1.98 1.64 -1.28 [-1.87, -0.69] 6.57
Hanna et al., 2008 138 -2.1 2.61 145 -1.5 2.38 -0.60 [-1.18, -0.02] 6.62
Heidari et al., 2019 43 -5.2 1.91 47 -3.45 1.5 -1.75 [-2.46, -1.04] 5.82
Saxena et al., 2024 15 -5 1.23 15 -3.14 1 -1.86 [-2.66, -1.06] 5.26
Shokri et al., 2021 52 -4.2 1.83 51 -2.9 1.56 -1.30 [-1.96, -0.64] 6.13
Simpson 2001 6 -2 .52 5 -.5 .52 -1.50 [-2.12, -0.88] 6.38
Tesfaye et al., 2024 37 -2.46 2.12 42 -2.09 1.46 -0.37 [-1.16, 0.42] 5.30
Vasudevan et al., 2014 14 -4 3.37 15 -4.13 3.37 0.13 [-2.32, 2.58] 1.14
Heterogeneity: τ² = 0.16, I² = 54.55%, H² = 2.20 -1.20 [-1.59, -0.81]
Test of θi = θj: Q(7) = 15.40, p = 0.03
Test of θ = 0: z = -6.09, p = 0.00
PHN
Huang et al., 2018 58 -3.8 1.31 58 -2.9 1.21 -0.90 [-1.36, -0.44] 7.42
Huang et al., 2021 96 -4.8 1.32 97 -3.5 1.4 -1.30 [-1.68, -0.92] 7.89
Saxena et al., 2021 20 -7.1 .97 20 -5.15 .84 -1.95 [-2.51, -1.39] 6.74
Tao et al., 2025 67 -4.25 1.51 67 -2.8 1.35 -1.45 [-1.93, -0.97] 7.25
Wu et al., 2025 49 -4.47 1.64 49 -3.94 1.49 -0.53 [-1.15, 0.09] 6.36
Yang et al., 2025 33 -4.22 .98 33 -2.68 .98 -1.54 [-2.01, -1.07] 7.33
Heterogeneity: τ² = 0.13, I² = 67.37%, H² = 3.06 -1.29 [-1.64, -0.94]
Test of θi = θj: Q(5) = 15.32, p = 0.01
Test of θ = 0: z = -7.23, p = 0.00
TN
Lemos et al., 2008 12 -6 1.39 12 -3.6 1.47 -2.40 [-3.54, -1.26] 3.63
Heterogeneity: τ² = 0.00, I² = .%, H² = . -2.40 [-3.54, -1.26]
Test of θi = θj: Q(0) = 0.00, p = .
Test of θ = 0: z = -4.11, p = 0.00
Overall -1.27 [-1.55, -0.99]
Heterogeneity: τ² = 0.22, I² = 69.11%, H² = 3.24
Test of θi = θj: Q(17) = 55.04, p = 0.00
Test of θ = 0: z = -8.93, p = 0.00
Test of group differences: Qb(3) = 4.30, p = 0.23
-6 -4 -2 0 2
Random-effects DerSimonian–Laird model

D

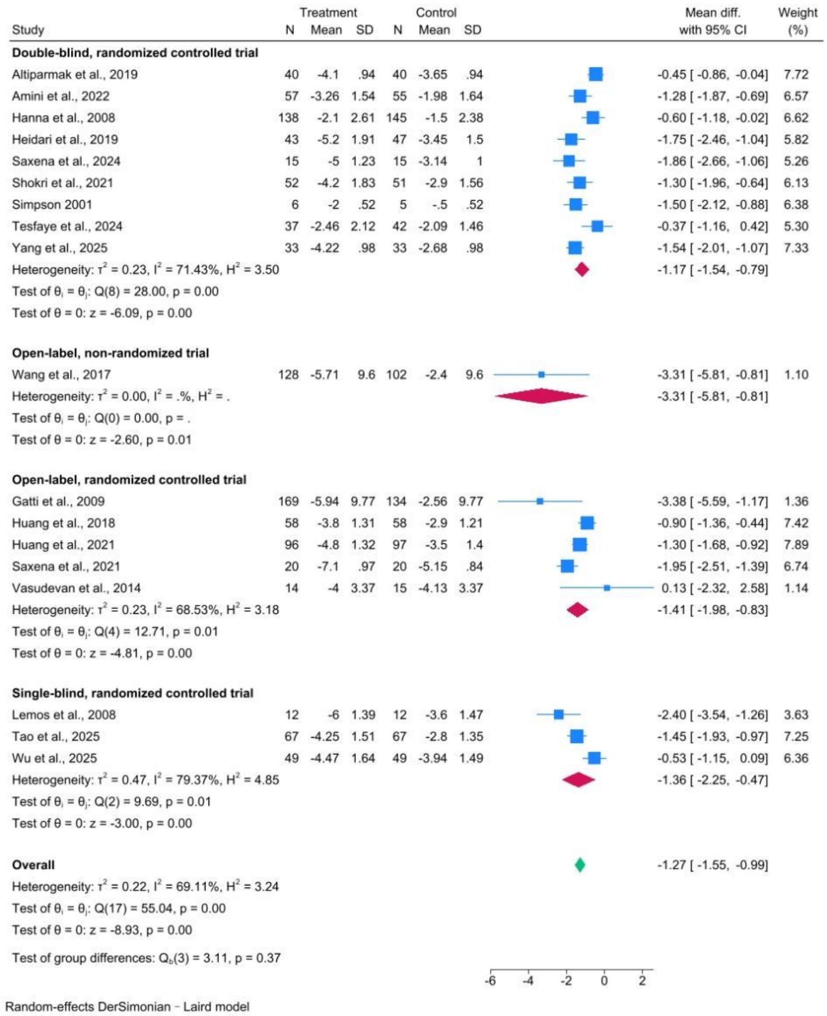

Figure S1. Subgroup analyses of the effects of gabapentinoid combination therapy versus monotherapy on the change of average pain scores from baseline in patients with neuropathic pain. Forest plot of average pain scores by class of combination therapy (A), monotherapy agent (B), disease (C), and study design (D).

A

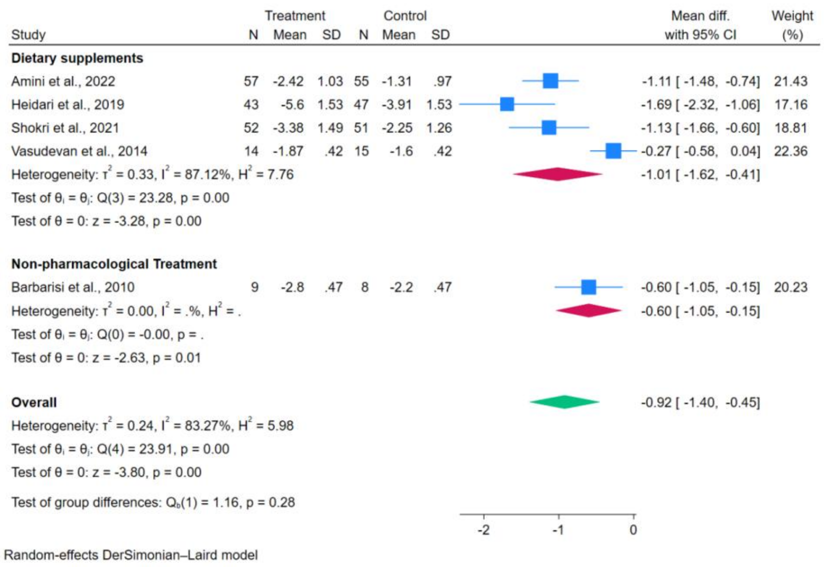
Treatment
Control
Study
N Mean SD N Mean SD
Mean diff. with 95% CI
Weight (%)
Dietary supplements
Amini et al., 2022 57 -2.42 1.03 55 -1.31 .97 -1.11 [-1.48, -0.74] 21.43
Heidari et al., 2019 43 -5.6 1.53 47 -3.91 1.53 -1.69 [-2.32, -1.06] 17.16
Shokri et al., 2021 52 -3.38 1.49 51 -2.25 1.26 -1.13 [-1.66, -0.60] 18.81
Vasudevan et al., 2014 14 -1.87 .42 15 -1.6 .42 -0.27 [-0.58, 0.04] 22.36
Heterogeneity: τ^2 = 0.33, I^2 = 87.12%, H^2 = 7.76 -1.01 [-1.62, -0.41]
Test of $\theta_i = \theta_j$: Q(3) = 23.28, p = 0.00
Test of θ = 0: z = -3.28, p = 0.00
Non-pharmacological Treatment
Barbarisi et al., 2010 9 -2.8 .47 8 -2.2 .47 -0.60 [-1.05, -0.15] 20.23
Heterogeneity: τ^2 = 0.00, I^2 = .%, H^2 = . -0.60 [-1.05, -0.15]
Test of $\theta_i = \theta_j$: Q(0) = -0.00, p = .
Test of θ = 0: z = -2.63, p = 0.01
Overall -0.92 [-1.40, -0.45]
Heterogeneity: τ^2 = 0.24, I^2 = 83.27%, H^2 = 5.98
Test of $\theta_i = \theta_j$: Q(4) = 23.91, p = 0.00
Test of θ = 0: z = -3.80, p = 0.00
Test of group differences: $Q_b(1)$ = 1.16, p = 0.28
-2
-1
0
Random-effects DerSimonian–Laird model

B

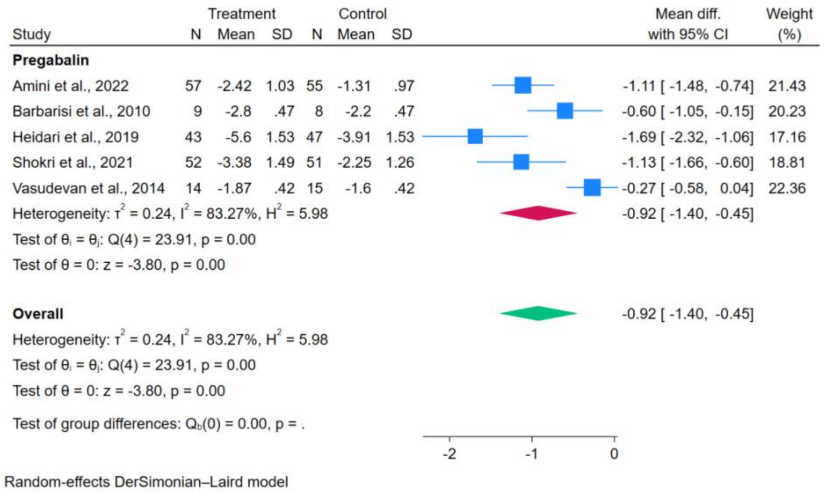
Treatment
Control
Study
N Mean SD N Mean SD
Mean diff. with 95% CI
Weight (%)
Pregabalin
Amini et al., 2022 57 -2.42 1.03 55 -1.31 .97 -1.11 [-1.48, -0.74] 21.43
Barbarisi et al., 2010 9 -2.8 .47 8 -2.2 .47 -0.60 [-1.05, -0.15] 20.23
Heidari et al., 2019 43 -5.6 1.53 47 -3.91 1.53 -1.69 [-2.32, -1.06] 17.16
Shokri et al., 2021 52 -3.38 1.49 51 -2.25 1.26 -1.13 [-1.66, -0.60] 18.81
Vasudevan et al., 2014 14 -1.87 .42 15 -1.6 .42 -0.27 [-0.58, 0.04] 22.36
Heterogeneity: τ^2 = 0.24, I^2 = 83.27%, H^2 = 5.98 -0.92 [-1.40, -0.45]
Test of $\theta_i = \theta_j$: Q(4) = 23.91, p = 0.00
Test of θ = 0: z = -3.80, p = 0.00
Overall -0.92 [-1.40, -0.45]
Heterogeneity: τ^2 = 0.24, I^2 = 83.27%, H^2 = 5.98
Test of $\theta_i = \theta_j$: Q(4) = 23.91, p = 0.00
Test of θ = 0: z = -3.80, p = 0.00
Test of group differences: $Q_b(0)$ = 0.00, p = .
-2
-1
0
Random-effects DerSimonian–Laird model

C

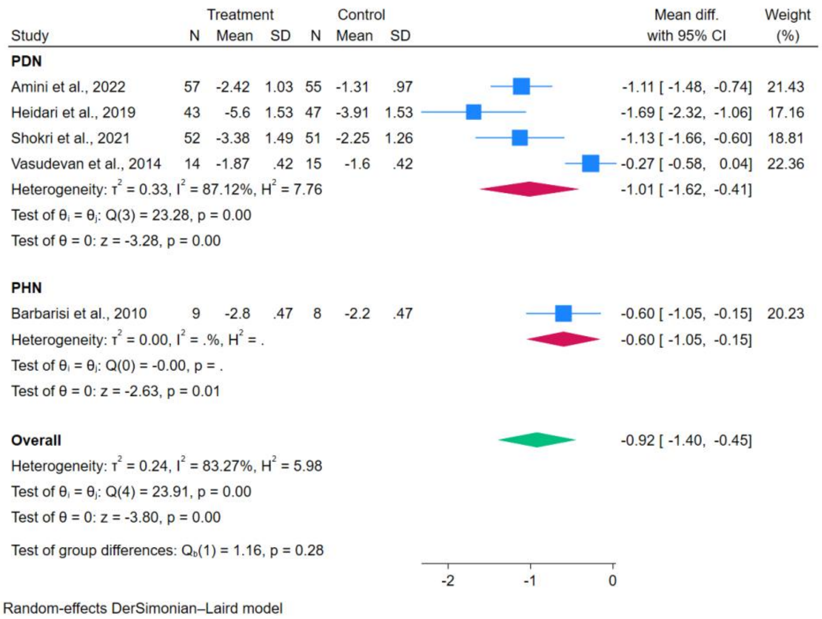

Figure S2. Subgroup analyses of the effects of gabapentinoid combination therapy versus monotherapy on sleep interference scores in patients with neuropathic pain. Forest plot of sleep interference scores by class of combination therapy (A), monotherapy agent (B), disease (C).

A

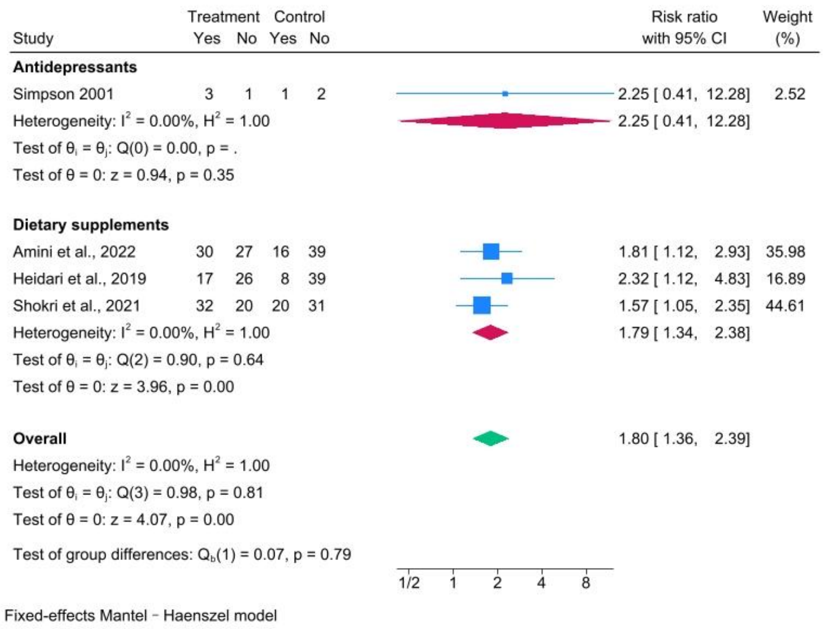
Study
Treatment Yes No
Control Yes No
Risk ratio with 95% CI
Weight (%)
Antidepressants
Simpson 2001 3 1 1 2 2.25 [0.41, 12.28] 2.52
Heterogeneity: I² = 0.00%, H² = 1.00 2.25 [0.41, 12.28]
Test of θi = θj: Q(0) = 0.00, p = .
Test of θ = 0: z = 0.94, p = 0.35
Dietary supplements
Amini et al., 2022 30 27 16 39 1.81 [1.12, 2.93] 35.98
Heidari et al., 2019 17 26 8 39 2.32 [1.12, 4.83] 16.89
Shokri et al., 2021 32 20 20 31 1.57 [1.05, 2.35] 44.61
Heterogeneity: I² = 0.00%, H² = 1.00 1.79 [1.34, 2.38]
Test of θi = θj: Q(2) = 0.90, p = 0.64
Test of θ = 0: z = 3.96, p = 0.00
Overall 1.80 [1.36, 2.39]
Heterogeneity: I² = 0.00%, H² = 1.00
Test of θi = θj: Q(3) = 0.98, p = 0.81
Test of θ = 0: z = 4.07, p = 0.00
Test of group differences: Qb(1) = 0.07, p = 0.79
1/2 1 2 4 8
Fixed-effects Mantel – Haenszel model

B

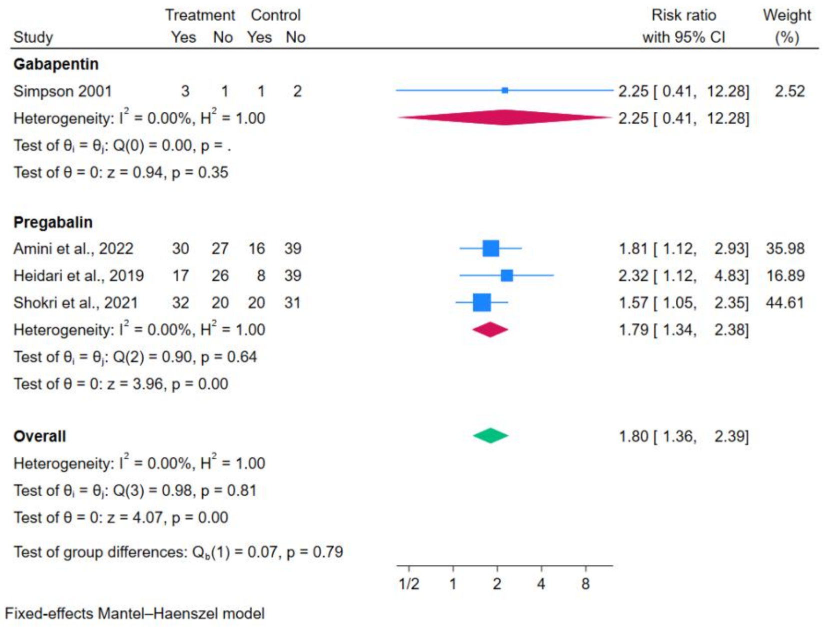
Study
Treatment Yes No
Control Yes No
Risk ratio with 95% CI
Weight (%)
Gabapentin
Simpson 2001 3 1 1 2 2.25 [0.41, 12.28] 2.52
Heterogeneity: I² = 0.00%, H² = 1.00 2.25 [0.41, 12.28]
Test of θi = θj: Q(0) = 0.00, p = .
Test of θ = 0: z = 0.94, p = 0.35
Pregabalin
Amini et al., 2022 30 27 16 39 1.81 [1.12, 2.93] 35.98
Heidari et al., 2019 17 26 8 39 2.32 [1.12, 4.83] 16.89
Shokri et al., 2021 32 20 20 31 1.57 [1.05, 2.35] 44.61
Heterogeneity: I² = 0.00%, H² = 1.00 1.79 [1.34, 2.38]
Test of θi = θj: Q(2) = 0.90, p = 0.64
Test of θ = 0: z = 3.96, p = 0.00
Overall 1.80 [1.36, 2.39]
Heterogeneity: I² = 0.00%, H² = 1.00
Test of θi = θj: Q(3) = 0.98, p = 0.81
Test of θ = 0: z = 4.07, p = 0.00
Test of group differences: Qb(1) = 0.07, p = 0.79
1/2 1 2 4 8
Fixed-effects Mantel–Haenszel model

C

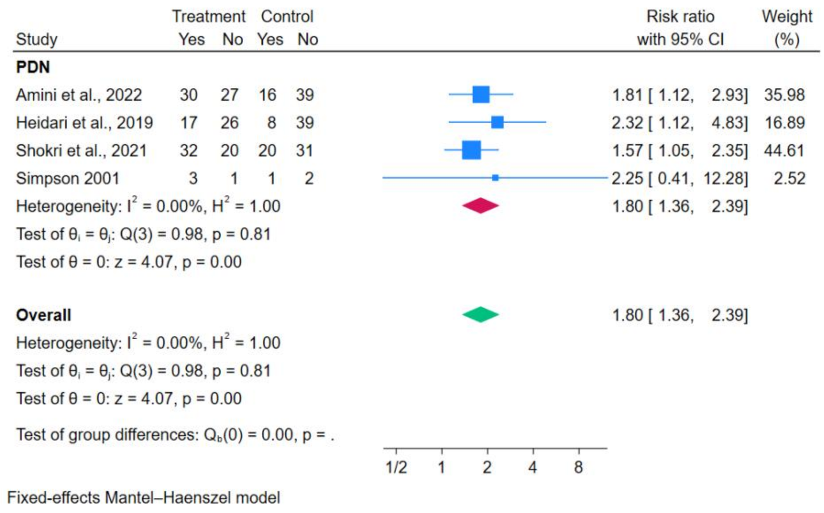

Figure S3. Subgroup analyses of the effects of gabapentinoid combination therapy versus monotherapy on Patient Global Impression of Change (PGIC) in patients with neuropathic pain. Forest plot of PGIC by class of combination therapy (A), monotherapy agent (B), disease (C).

A

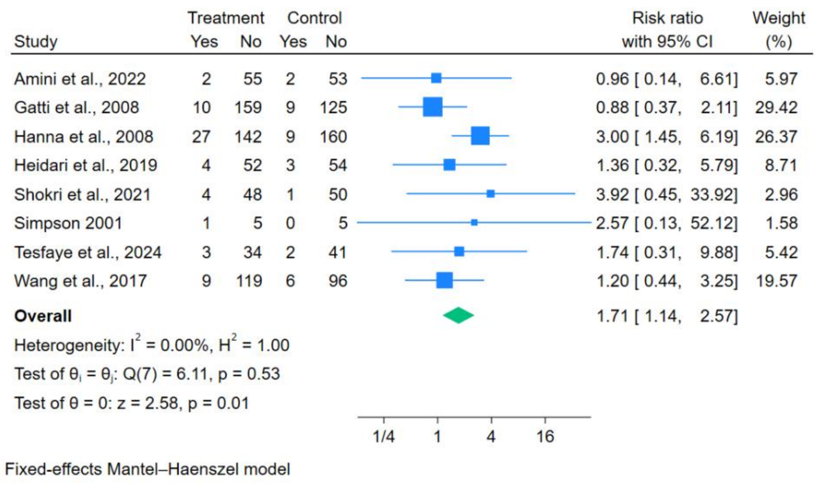

B

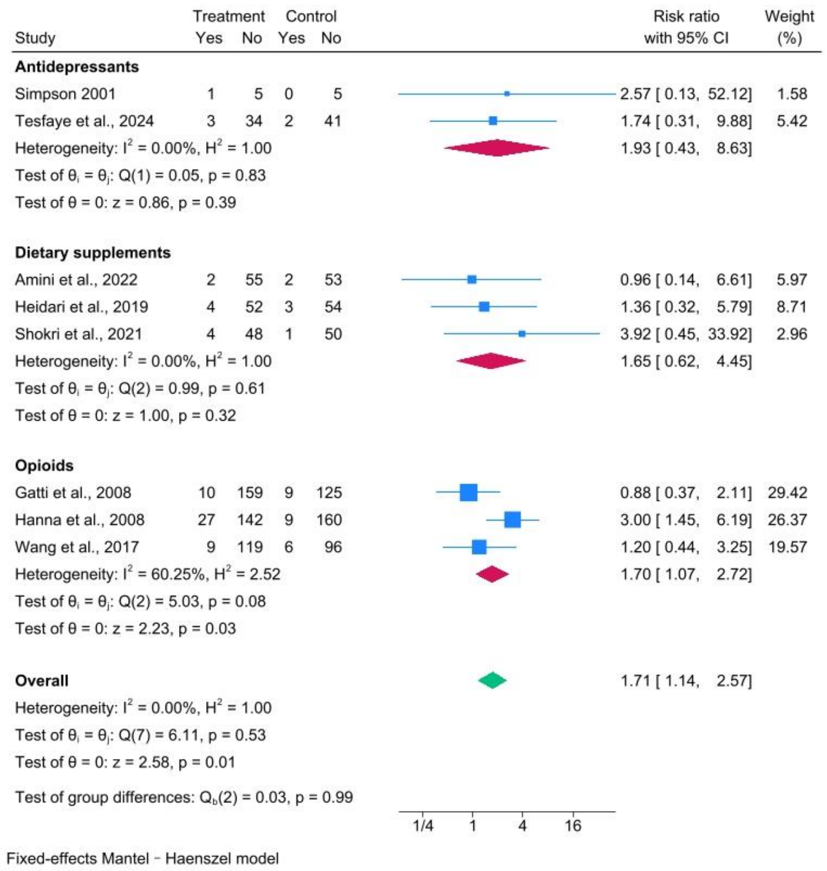

Figure S4. Risk of discontinuations due to adverse events between gabapentinoid combination therapy versus monotherapy. Forest plot of discontinuations due to adverse events (A). Subgroup analyses of the risk of discontinuations due to adverse events by the class of combination therapy (B).

A

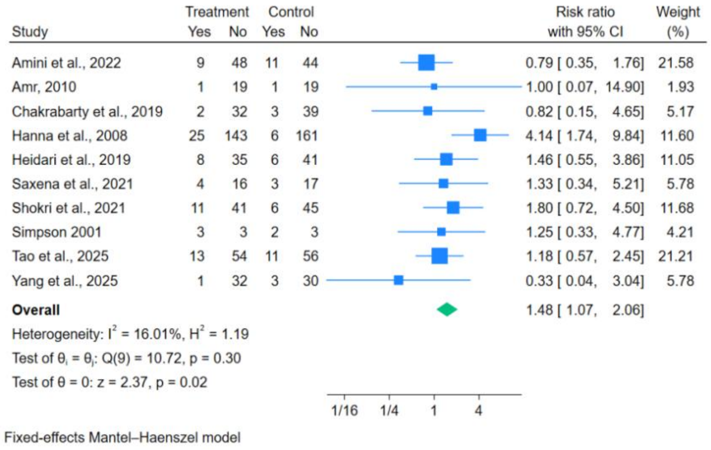

B

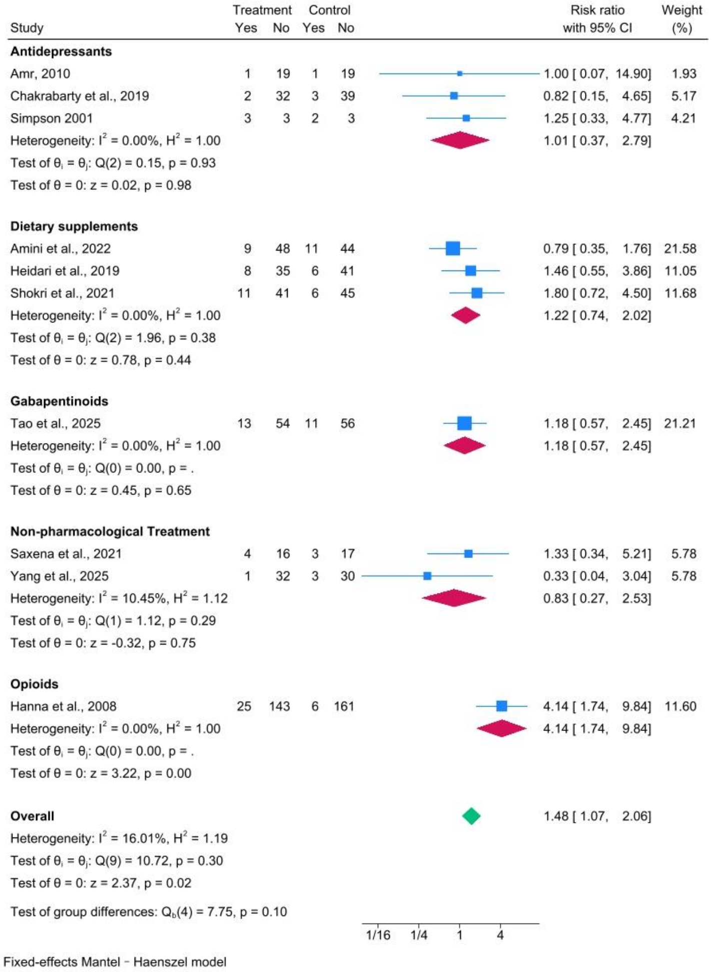

Figure S5. Risk of dizziness between gabapentinoid combination therapy versus monotherapy. Forest plot of the risk of dizziness (A). Subgroup analyses of the risk of dizziness by the class of combination therapy (B).

A

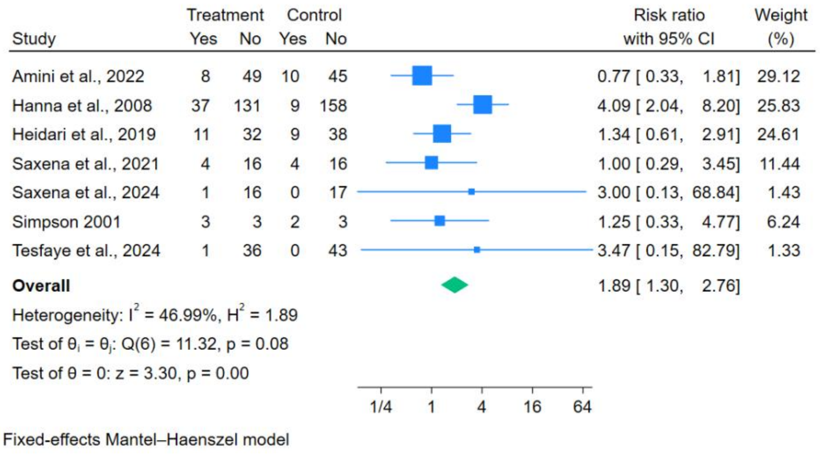

B

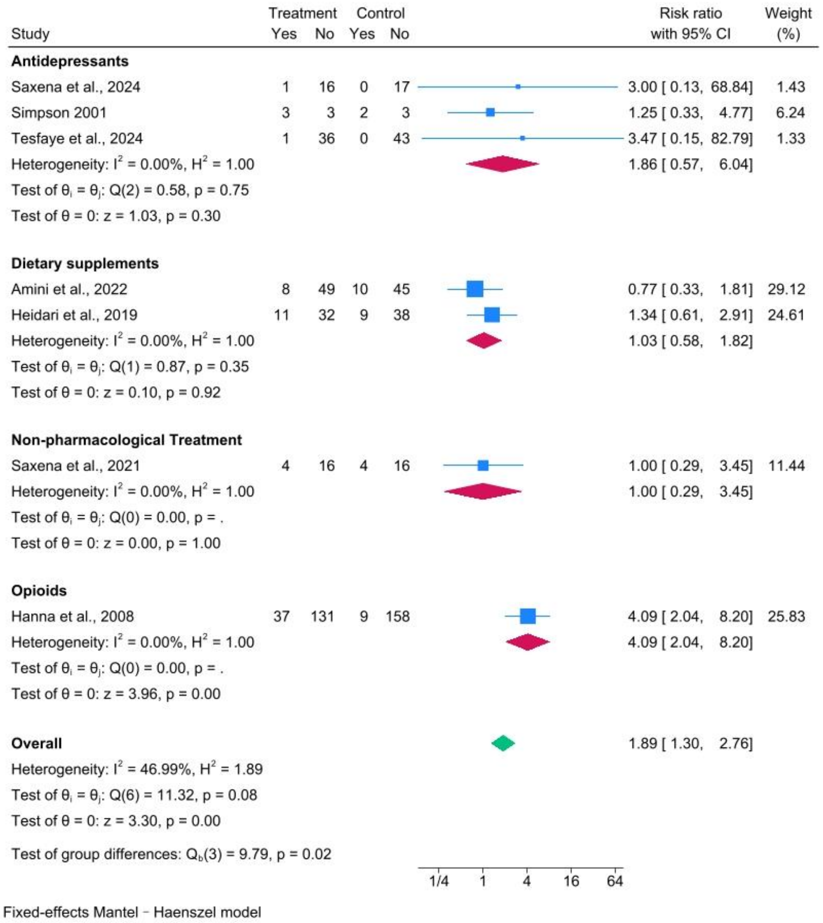

Figure S6. Risk of somnolence between gabapentinoid combination therapy versus monotherapy. Forest plot of the risk of somnolence (A). Subgroup analyses of the risk of somnolence by the class of combination therapy (B).

A

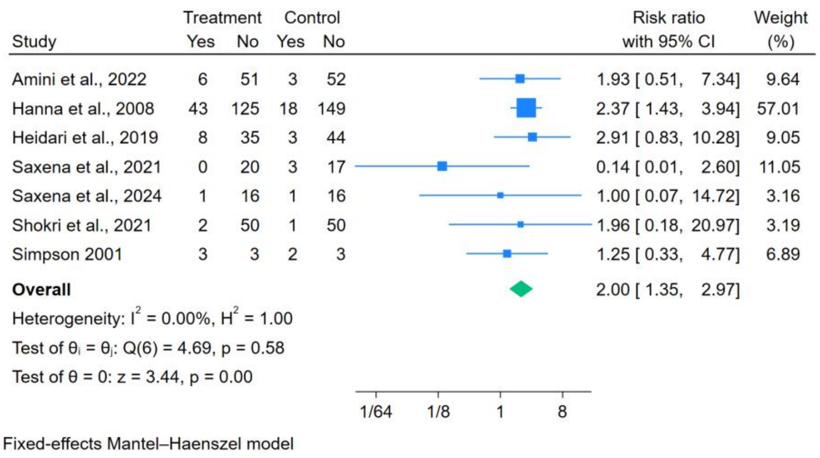

B

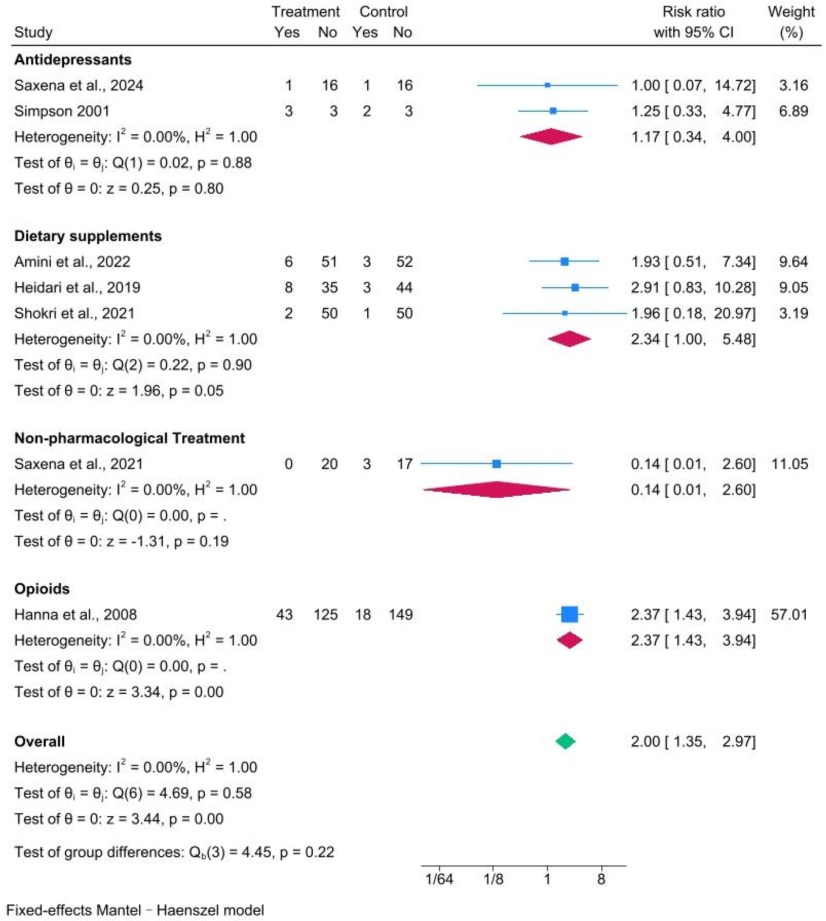

Figure S7. Risk of nausea between gabapentinoid combination therapy versus monotherapy. Forest plot of the risk of nausea (A). Subgroup analyses of the risk of nausea by the class of combination therapy (B).

A

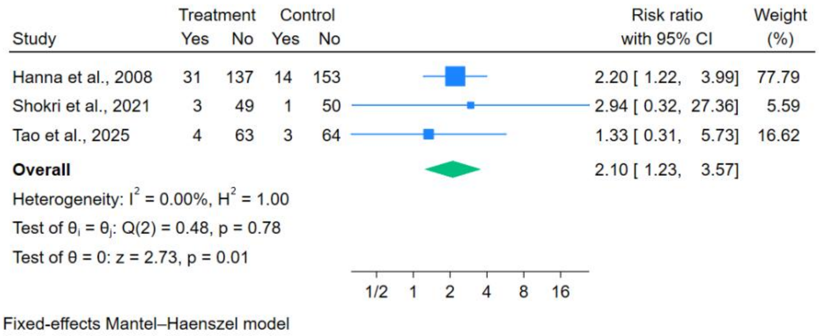

B

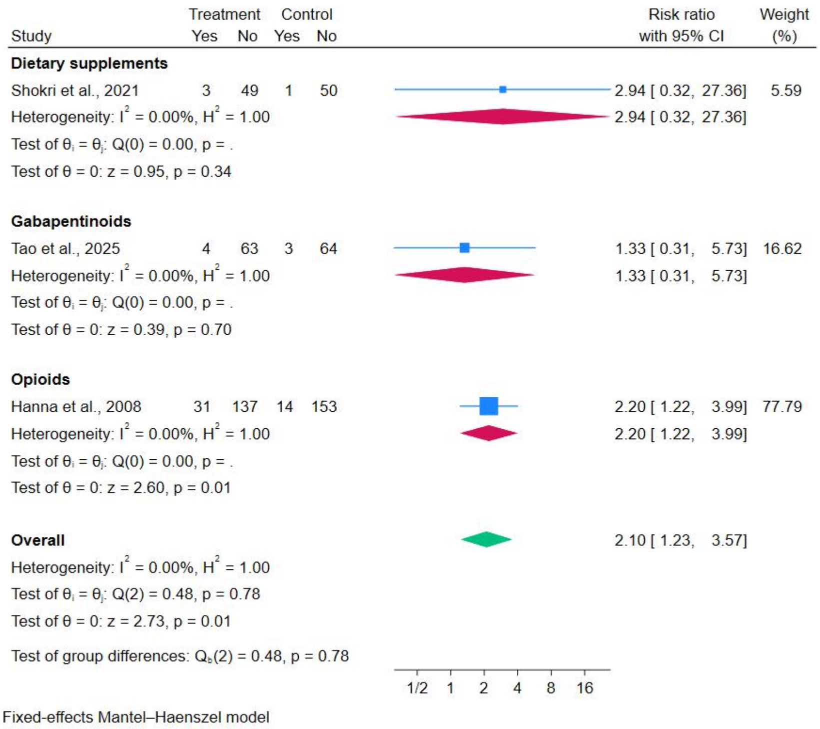

Figure S8. Risk of fatigue between gabapentinoid combination therapy versus monotherapy. Forest plot of the risk of fatigue (A). Subgroup analyses of the risk of fatigue by the class of combination therapy (B).

A

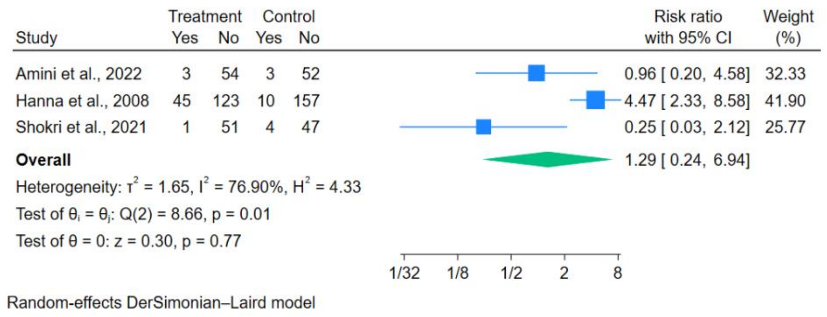

B

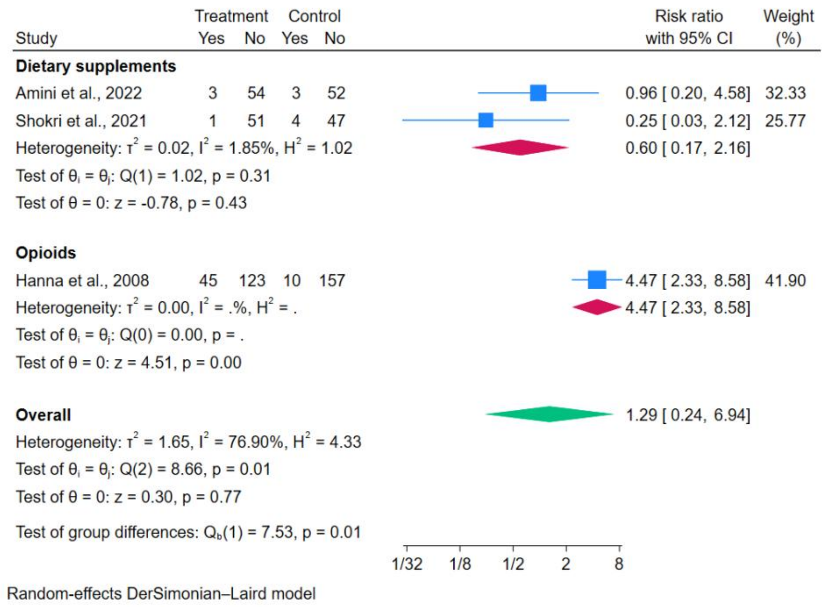

Figure S9. Risk of constipation between gabapentinoid combination therapy versus monotherapy. Forest plot of the risk of constipation (A). Subgroup analyses of the risk of constipation by the class of combination therapy (B).

A

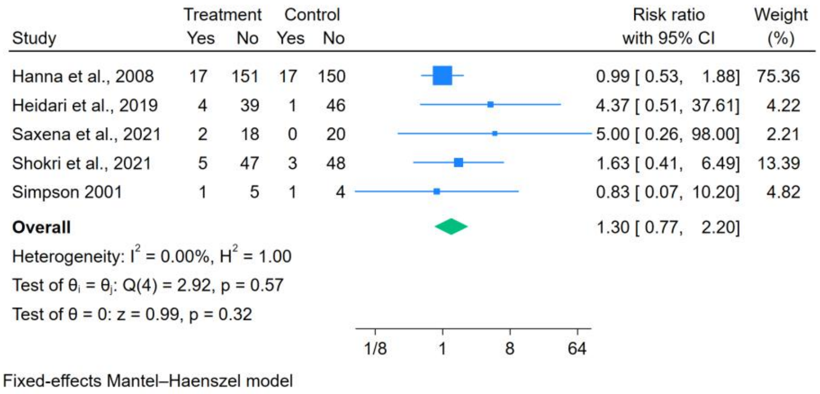

B

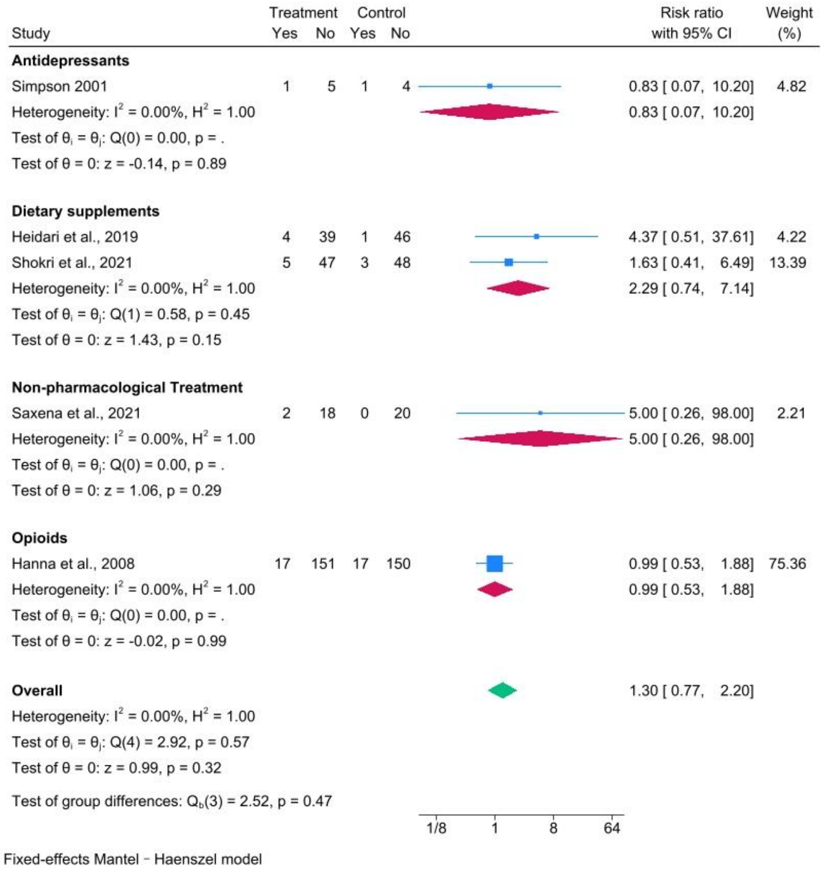

Figure S10. Risk of headache between gabapentinoid combination therapy versus monotherapy. Forest plot of the risk of headache (A). Subgroup analyses of the risk of headache by the class of combination therapy (B).

A

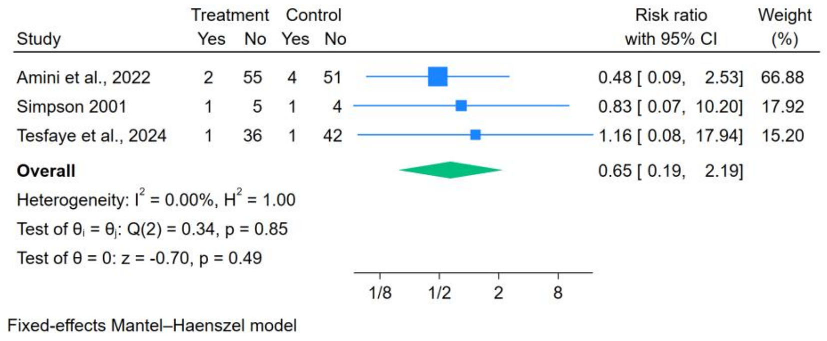

B

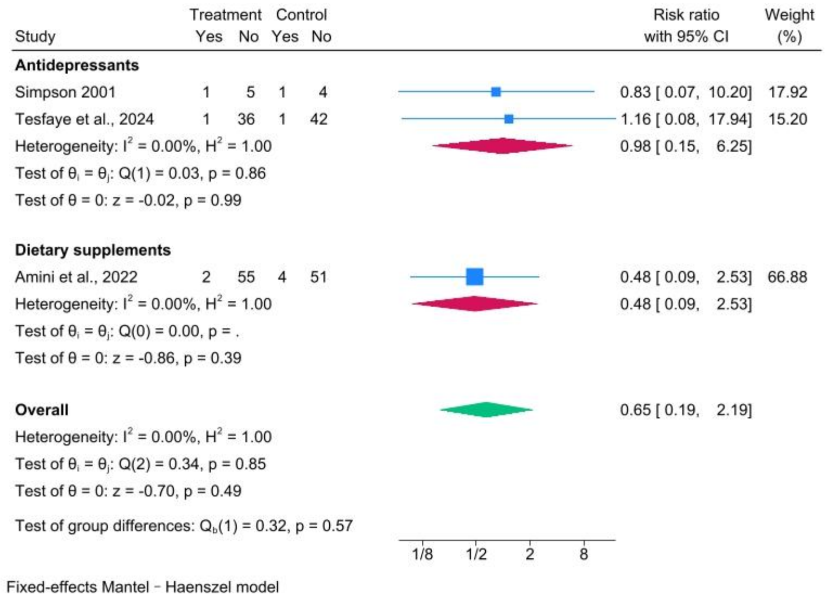

Figure S11. Risk of diarrhea between gabapentinoid combination therapy versus monotherapy. Forest plot of the risk of diarrhea (A). Subgroup analyses of the risk of diarrhea by the class of combination therapy (B).

A

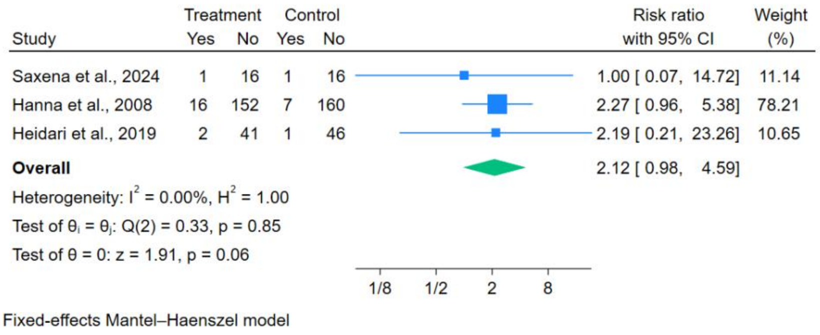

B

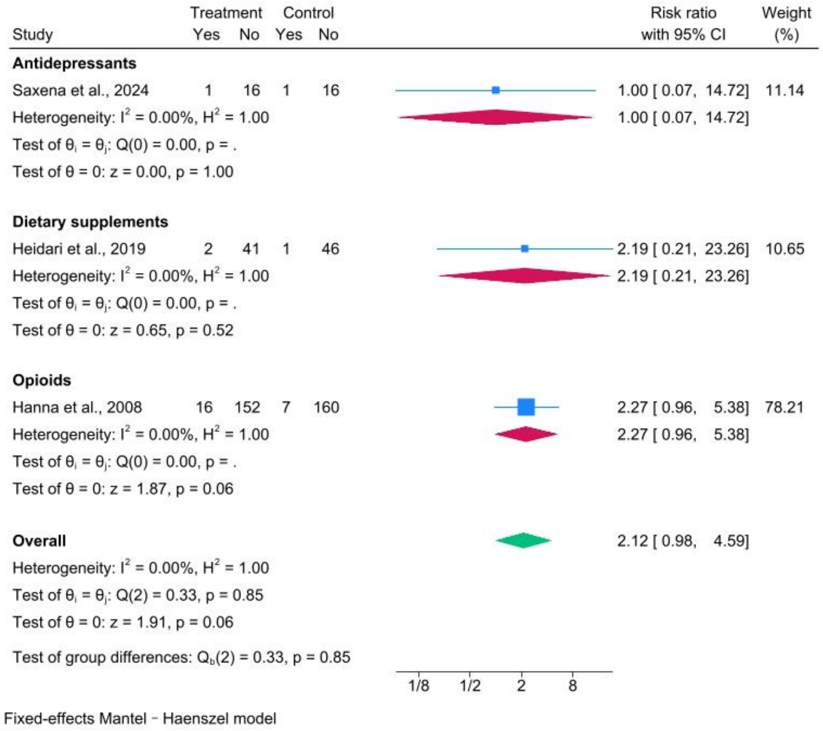

Figure S12. Risk of vomiting between gabapentinoid combination therapy versus monotherapy. Forest plot of the risk of vomiting (A). Subgroup analyses of the risk of vomiting by the class of combination therapy (B).

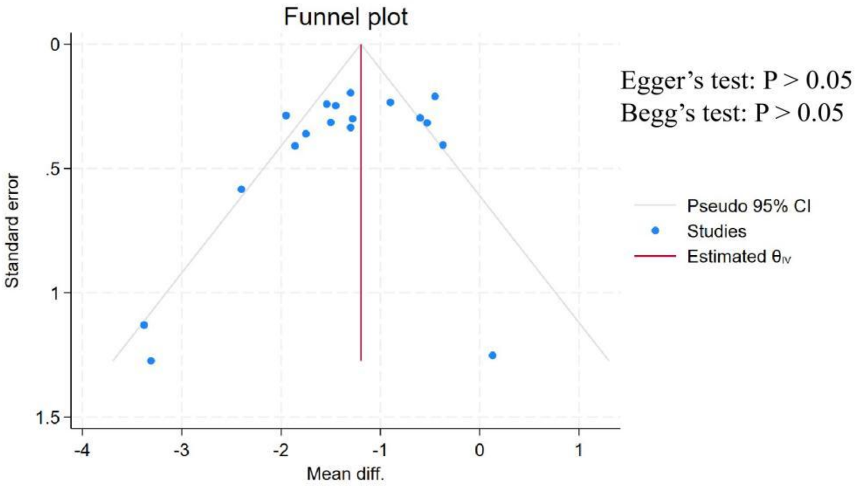

Figure S13. Funnel plot for the assessment of publication bias.

A

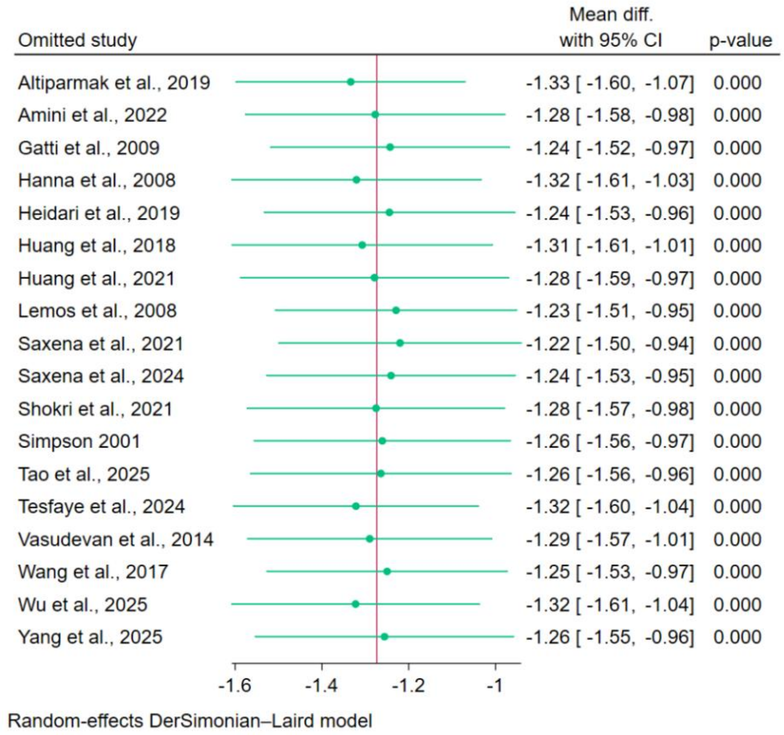

B

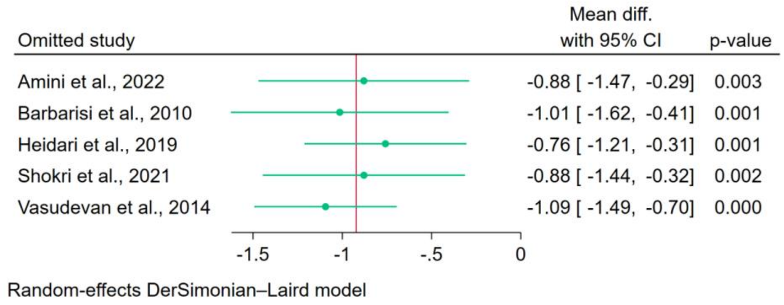

C

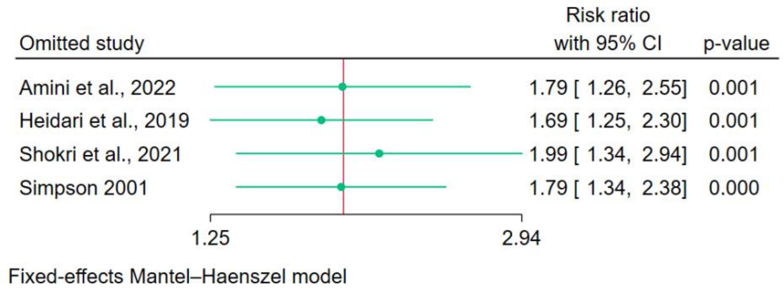

Figure S14. Sensitivity analyses by leave-one-out method. Sensitivity analyses for average pain scores change from baseline (A), sleep interference scores (B), and Patient Global Impression of Change (PGIC) (C).

Supplementary Appendix S1: Supplementary Methods. Search Strategy of Pubmed (A), Embase (B), Web of Sciense (C) and Cochrane Library (D).

A

#1 "Neuralgia"[Mesh]

#2 (((((((((((((neuropathic pain[Title/Abstract]) OR (diabetic neuropathy[Title/Abstract])) OR (diabetic peripheral neuropathy[Title/Abstract])) OR (painful polyneuropathy[Title/Abstract])) OR (diabetic polyneuropathy[Title/Abstract])) OR (postherpetic neuralgia[Title/Abstract])) OR (peripheral nerve injury[Title/Abstract])) OR (central post stroke pain[Title/Abstract])) OR (low back pain[Title/Abstract])) OR (multiple sclerosis[Title/Abstract])) OR (phantom limb pain[Title/Abstract])) OR (radiculopathy[Title/Abstract])) OR (small fibre neuropathy[Title/Abstract])) OR (spinal cord injury[Title/Abstract])) OR (trigeminal neuralgia[Title/Abstract])

#3 #1 OR #2

#4 (((gabapentinoids[Title/Abstract]) OR (pregabalin[Title/Abstract])) OR (gabapentin[Title/Abstract])) OR (mirogabalin[Title/Abstract])

#5 ((((((((combination[Title/Abstract]) OR (combining[Title/Abstract])) OR (combined[Title/Abstract])) OR (add-on[Title/Abstract])) OR (plus[Title/Abstract])) OR (addition[Title/Abstract])) OR (adding[Title/Abstract])) OR (adjuvant[Title/Abstract])) OR (co-administration[Title/Abstract])

#6 (((trial[Title/Abstract]) OR (double-blind[Title/Abstract])) OR (placebo[Title/Abstract])) OR (clinical trial[Publication Type])

#7 #3 AND #4 AND #5 AND #6

B

#1 neuralgia:ti,ab,kw OR 'neuropathic pain':ti,ab,kw OR 'diabetic neuropathy':ti,ab,kw OR 'diabetic peripheral neuropathy':ti,ab,kw OR 'painful polyneuropathy':ti,ab,kw OR 'diabetic polyneuropathy':ti,ab,kw OR 'postherpetic neuralgia':ti,ab,kw OR 'peripheral nerve injury':ti,ab,kw OR 'central post stroke pain':ti,ab,kw OR 'low back pain':ti,ab,kw OR 'multiple sclerosis':ti,ab,kw OR 'phantom limb pain':ti,ab,kw OR radiculopathy:ti,ab,kw OR 'small fibre neuropathy':ti,ab,kw OR 'spinal cord injury':ti,ab,kw OR 'trigeminal neuralgia':ti,ab,kw

#2 gabapentinoids:ti,ab,kw OR pregabalin:ti,ab,kw OR gabapentin:ti,ab,kw OR mirogabalin:ti,ab,kw

#3 combination:ti,ab,kw OR combining:ti,ab,kw OR combined:ti,ab,kw OR add-on:ti,ab,kw OR plus:ti,ab,kw OR addition:ti,ab,kw OR adding:ti,ab,kw OR adjuvant:ti,ab,kw OR co-administration:ti,ab,kw

#4 trial:ti,ab,kw OR double-blind:ti,ab,kw OR placebo:ti,ab,kw OR 'clinical trial':pt

#5 #1 AND #2 AND #3 AND #4

C

#1 ((((((((((((((AB=(neuralgia)) OR AB=(neuropathic pain)) OR AB=(diabetic neuropathy)) OR AB=(diabetic peripheral neuropathy)) OR AB=(painful polyneuropathy)) OR AB=(diabetic polyneuropathy)) OR AB=(postherpetic neuralgia)) OR AB=(peripheral nerve injury)) OR AB=(central post stroke pain)) OR AB=(low back pain)) OR AB=(multiple sclerosis)) OR AB=(phantom limb pain)) OR AB=(radiculopathy)) OR AB=(small fibre neuropathy)) OR AB=(spinal cord injury)) OR AB=(trigeminal neuralgia)

#2 (((AB=(gabapentinoids)) OR AB=(pregabalin)) OR AB=(gabapentin)) OR AB=(mirogabalin)

#3 ((((((((AB=(combination)) OR AB=(combining)) OR AB=(combined)) OR AB=(add-on)) OR AB=(plus)) OR AB=(addition)) OR AB=(adding)) OR AB=(adjuvant)) OR AB=(co-administration)

#4 ((ALL=(trial)) OR ALL=(double-blind)) OR ALL=(placebo)

#5 #1 AND #2 AND #3 AND #4

D

#1 MeSH descriptor: [Neuralgia] explode all trees

#2 (neuralgia):ti,ab,kw OR (neuropathic pain):ti,ab,kw OR (diabetic neuropathy):ti,ab,kw OR (diabetic peripheral neuropathy):ti,ab,kw OR (painful polyneuropathy):ti,ab,kw

#3 (diabetic polyneuropathy):ti,ab,kw OR (postherpetic neuralgia):ti,ab,kw OR (peripheral nerve injury):ti,ab,kw OR (central post stroke pain):ti,ab,kw OR (low back pain):ti,ab,kw

#4 (multiple sclerosis):ti,ab,kw OR (phantom limb pain):ti,ab,kw OR (radiculopathy):ti,ab,kw OR (small fibre neuropathy):ti,ab,kw OR (spinal cord injury):ti,ab,kw

#5 (trigeminal neuralgia):ti,ab,kw

#6 #1 OR #2 OR #3 OR #4 OR #5

#7 (gabapentinoids):ti,ab,kw OR (pregabalin):ti,ab,kw OR (gabapentin):ti,ab,kw OR (mirogabalin):ti,ab,kw

#8 (combination):ti,ab,kw OR (combining):ti,ab,kw OR (combined):ti,ab,kw OR (add-on):ti,ab,kw OR (plus):ti,ab,kw

#9 (addition):ti,ab,kw OR (adding):ti,ab,kw OR (adjuvant):ti,ab,kw OR (co-administration):ti,ab,kw

#10 #8 OR #9

#11 (trial):ti,ab,kw OR (double-blind):ti,ab,kw OR (placebo):ti,ab,kw OR (clinical trial):pt

#12 #6 AND #7 AND #10 AND #11
